# Adherence, Acceptability, and Sexual Health Outcomes of the Odeya App–Based Intervention for Sexual Distress in Women With Endometriosis: Randomized Controlled Mixed Methods Trial

**DOI:** 10.2196/86042

**Published:** 2026-02-19

**Authors:** Selina Marie Kronthaler, Eden Kosman, Tatjana Tissen-Diabaté, Elena Mühle, Luzia Weber- Schallauer, Therese Pross, Maria Margarete Karsten, Klaus Michael Beier, Laura Hatzler

**Affiliations:** 1 Institute of Sexology and Sexual Medicine Charité - Universitätsmedizin Berlin Berlin Germany; 2 Institute of Social Medicine, Epidemiology and Health Economics Charité – Universitätsmedizin Berlin Berlin Germany; 3 Department of Gynecology and Breast Center Charité – Universitätsmedizin Berlin Berlin Germany; 4 Department of Urology Charité - Universitätsmedizin Berlin Berlin Germany

**Keywords:** app-based intervention, digital health intervention, endometriosis, randomized controlled trial, sexual distress, sexual dysfunction

## Abstract

**Background:**

Evidence-based interventions effectively treat sexual dysfunctions. Up to 13.5% of women with gynecological conditions are affected, yet access to therapy is limited. Self-guided digital interventions may offer scalable, accessible first-line support.

**Objective:**

This randomized controlled mixed methods pilot trial evaluated adherence, acceptance, and safety of the Odeya app and changes in sexual and health outcomes among women with sexual dysfunctions and endometriosis.

**Methods:**

Following online and flyer-based recruitment, participants completed an online screening and were randomized to either an intervention group (IG) receiving 8 self-guided app modules targeting biopsychosocial aspects of sexuality or to a control group (CG) receiving routine care. Self-administered online questionnaires were completed at baseline (T0), midintervention (T1), postintervention (T2), and 6-month follow-up (T3). Standardized instruments assessed acceptance (Client Satisfaction Questionnaire-Internet [CSQ-I] and German mHealth App Usability Questionnaire [G-MAUQ]), safety (Inventory for the balanced assessment of Negative Effects of Psychotherapy-Online Intervention), sexual health (Female Sexual Distress Scale-Desire/Arousal/Orgasm [FSDS-DAO], Female Sexual Function Index-German version [FSFI-d], and Partnership Questionnaire), and overall health (Patient-Reported Outcome Measurement Information System-29-Item Profile, Beck Depression Inventory-II, and Generalized Anxiety Disorder-7). Adherence indicators included module completion, dropout rates, and symptom tracker use. Group differences were examined descriptively and using Cohen *d*. Qualitative data were collected through free-list questionnaires from dropouts (n=11) and interviews with completers (IG: n=3; CG: n=2).

**Results:**

A total of 60 women (mean age 31.12, SD 6.67 years) with confirmed or suspected endometriosis and sexual distress (FSDS-DAO score >18) were randomized to the IG (n=29) or CG (n=31). IG participants completed on average 61.2% (4.9/8) of modules; the dropout rate was 65.5% (19/29). Emotional strain, time demands, and technical issues were key barriers causing dropout, while persona-based stories facilitated engagement. Participants wished for more professional interaction. IG completers (n=10, 34.5%) showed lower baseline depression and anxiety but higher sexual distress. Satisfaction was high (CSQ-I=26.60; G-MAUQ=5.38). Although some adverse health changes were reported, findings indicate safety. FSDS-DAO scores decreased in both groups, with mean reductions from baseline of −10.39, −12.61, and −14.98 in the IG and −3.68, −14.83, and −6.92 in the CG from T1 to T3, respectively. Moderate to large between-group effects favoring the IG were observed at T1 (*d*=−0.66) and T3 (*d*=−0.79). Sexual function (FSFI-d) improved only in the IG (T1-T3: *d*=0.16-1.00). Qualitative findings highlighted rediscovery of positive sexual experiences, improved communication, and increased openness. Both groups reported improvements in anxiety, depression, and physical functioning, with additional gains in emotion regulation, distress reduction, and body awareness reported in the IG. Women emphasized symptom complexity and a need for more professional guidance.

**Conclusions:**

The self-guided intervention was well accepted and showed preliminary improvements among completers. Adherence and sustained engagement seemed shaped by baseline psychosocial health, pointing to a need for tailored adaptations and larger confirmatory trials.

**Trial Registration:**

German Clinical Trials Register DRKS00034351; https://drks.de/search/en/trial/DRKS00034351

## Introduction

### Overview

Sexual health is a fundamental aspect of overall well-being and quality of life [1-3]. Sexual dysfunctions, such as problems with sexual desire, arousal, orgasm, or pain, accompanied by clinically relevant distress, as defined by the *ICD-11* (*International Classification of Diseases, 11th Revision*) [4], were reported by 17.5% of women in a representative German study [5]. The biopsychosocial risk factors include relationship-related difficulties, poor mental health, chronic conditions, cultural factors, and lack of knowledge and experience [6-10].

Among individuals with chronic diseases, including gynecological conditions such as endometriosis, sexual health issues are particularly pronounced [11]. In a recent representative German study, 75.2% of those with chronic conditions reported problems in sexual function, and 19.3% met *ICD-11* criteria for sexual dysfunction, with 2.56-fold higher odds compared to individuals without chronic conditions [12]. Among all chronic condition groups, women with gynecological conditions showed the highest prevalence of problems in sexual function (84.3%), and 13.5% of these women met criteria for sexual dysfunction with distress [12]. Chronic diseases frequently contribute to sexual dysfunction through physical, hormonal, psychological, and treatment-related factors [13-15], with downstream impacts on mental health, relationship satisfaction, and health care costs [16-19].

Endometriosis affects 6.8% of women worldwide [20] and substantially impairs sexual health and quality of life [21,22]. The association between endometriosis and sexual dysfunction is well-documented, with many reporting sexual pain, decreased sexual satisfaction, and overall reduced sexual functioning [11,23]. Surgical interventions may further worsen outcomes [24]. Care access is hindered by shame and stigma, insufficient awareness, high costs, and gaps in provider training [12,25-30].

Effective treatment for sexual dysfunction with distress requires a personalized, multimodal, interdisciplinary approach addressing the individual’s set of biopsychosocial etiological factors [10,31-36]. Recommended strategies combine somatic interventions (eg, pelvic floor therapy and hormonal and medical treatments) [37-40] with sex and couples therapy (eg, sensate focus and communication training), educational components (eg, psychoeducation on anatomy and physiology) [33,41], and lifestyle-based strategies such as adapted physical activity [42,43]. Furthermore, evidence supports strengthening the mind-body integration through exercises on body perception, mindfulness, and reflective techniques [41,44-46]. Underlying mental health and somatic conditions should always be addressed in interdisciplinary approaches, ideally by a multidisciplinary team trained in sexual medicine [35,47].

Despite high prevalence, sexual dysfunctions remain underreported and undertreated in Germany [12]. Persistent access barriers—including limited specialist availability, long waiting lists, and regional disparities—contrast with strong interest in digital interventions such as app- or web-based programs with exercises and educational content on sexual health [12,29,48].

Digital self-help interventions can help overcome these barriers [16], offering accessibility, anonymity, and cost-effectiveness [49-51]. Online treatments—especially cognitive-behavioral and mindfulness-based programs—have shown moderate to large effects on sexual function and satisfaction [50,52-56]. In 2019, the German Digital Healthcare Act introduced a regulatory framework for the approval and reimbursement of software as a medical device, referred to as digital health applications (DiGAs) [57]. Currently, 2 sexual health-related DiGAs are approved and permanently listed in the registry of the German competent authority (Bundesinstitut für Arzneimittel und Medizinprodukte [BfArM]): HelloBetter Vaginismus for vaginismus [50,58] and Kranus Edera for erectile dysfunction [59]. Additional DiGAs targeting urogenital health include Endo App for endometriosis symptom management [60,61] and Kranus Lutera for lower tract symptoms [62]. Negotiated 90-day prices averaged €221.09 (US $258.70) [63], with full statutory reimbursement.

Patient-centered digital intervention development [64] has demonstrated high user satisfaction [65,66] and positive outcomes across diverse populations, including cancer [67-69], mental health conditions such as depression and anxiety [70-72], and chronic pain [73-75]. Yet adherence and engagement remain challenging—especially in self-guided formats [76-82]—and effects on engagement are mixed [73,83-89]. A major gap persists in digital interventions addressing sexual distress in women with gynecological conditions such as endometriosis, underscoring the need for tailored, patient-centered solutions [90]. Existing digital tools rarely address sexuality as a relational resource; insights from syndyastic sex therapy may offer a useful framework for future digital models [91].

### Objective

This study evaluated the pilot implementation of a self-guided smartphone app intervention (Odeya) in women with sexual distress and diagnosed or suspected endometriosis. The Odeya app was developed using evidence-based content for patients with sexual dysfunctions in conjunction with clinically relevant distress and was tailored, within a patient-centered framework, to the specific needs of women with endometriosis. The primary objectives were to assess (1) adherence, (2) acceptance, and (3) safety. The secondary objective was to exploratorily examine effects on sexual and overall health-related outcomes using a mixed methods design.

## Methods

### Trial Design

A preceding patient-centered, iterative development phase informed the intervention and functioned as a preuse acceptability assessment of burden, usability, relevance, and coherence (E Kosman, MSc, et al, unpublished data, 2026). Building on this foundation, this open-label, 2-arm pilot randomized controlled trial (July 2024 to July 2025) used an expansion-type mixed methods design, collecting quantitative and qualitative data to enrich and explain emerging findings (see Multimedia Appendix 1 [6-8,16,33,41,45,47,64,65,92-127] for design rationale and prestudy procedures) [128]. Longitudinal self-reported data were collected in the intervention group (IG) and control group (CG) at baseline (T0) after randomization, peritreatment (T1; week 5 in the CG/after completing module 5 in the IG), posttreatment (T2; week 8 in the CG/after completing module 8 in the IG), and at 6-month follow-up (T3). The IG received Odeya app access; the CG was allowed to use existing treatment options within the health care system (treatment as usual) and offered later access. The trial was preregistered (German Clinical Trials Register: DRKS00034351) [129] and adhered to Consolidated Standards of Reporting Trials of Electronic and Mobile Health Applications and Online TeleHealth checklist (Multimedia Appendix 2) [130], and National Institutes of Health best practice guidelines for mixed methods research in the health sciences [131].

### Participants

Participants had to meet the following criteria to be eligible: (1) sufficient understanding of the German language, (2) being at least 18 years of age, (3) a physician-suspected or confirmed diagnosis of endometriosis or adenomyosis, (4) clinically relevant sexual distress (Female Sexual Distress Scale-Desire/Arousal/Orgasm [FSDS-DAO] >18), and (5) owning a smartphone (iOS or Android). The exclusion criteria were (1) current severe depression (Beck Depression Inventory-II ≥29) [92], (2) current severe anxiety (Generalized Anxiety Disorder 7-item ≥15) [93], (3) suicidal tendencies in the last 5 years, (4) current symptoms of posttraumatic stress disorder (PTSD), (5) substance dependence in the last 2 years, (6) current psychosis or dissociative symptoms, and (7) current pregnancy. If the online screening responses indicated the presence of potential PTSD or substance dependence, the participants were invited via email to take part in an additional telephone screening interview. The International Trauma Questionnaire [132] was used to assess PTSD, and the relevant section of the Structured Clinical Interview for the *DSM-IV* (*Diagnostic and Statistical Manual of Mental Disorders, Fourth Edition*) [133] was used to assess substance use.

### Intervention

The Odeya intervention was a self-guided smartphone app intervention developed to address sexual distress in women with endometriosis, for both single and partnered users.

**Figure 1:**
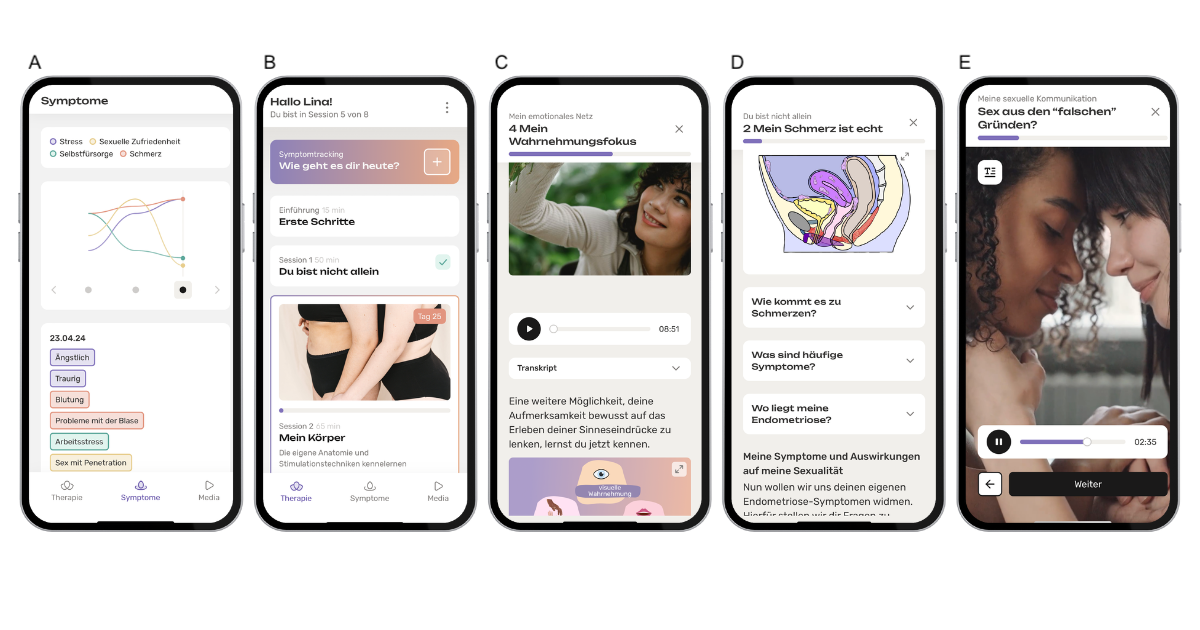
Interface of the Odeya intervention. Five smartphone screens are shown from left to right: (A) symptom-tracking overview displaying symptom levels (upper section) and present symptoms for a single entry (lower section); (B) overview of intervention modules including modules 1 and 2; (C) example of module content with graphics and an audio exercise; (D) example of psychoeducational content presented as text; and (E) example of psychoeducational content presented as video.

It was developed at the Institute of Sexology and Sexual Medicine, Charité–Universitätsmedizin Berlin, within the Berlin Institute of Health Digital Health Accelerator program, in collaboration with Hybrid Heroes GmbH, using a patient-centered, iterative process (E Kosman, MSc, et al, unpublished data, 2026). The intervention comprises 8 self-guided modules and a symptom-tracking tool, intended for completion over 12 weeks with a 4-week buffer. Modules unlock weekly, each taking ~60 minutes plus 15-60 minutes of exercises. Users can pause and resume at any time. See Table 1 and Figure 1 for a list of module topics and examples of user interface screens. Delivery was multimodal (text, video, audio, graphics, or interactive tasks) with exercises such as pelvic floor training, guided body-based activities, mindfulness, and sensate focus for solo or partnered practice. Participants in relationships were encouraged to involve their partners, while equivalent solo options were provided. The symptom-tracking tool monitors 4 domains (body, mind, social, and sexuality) using 1-10 ratings for pain, stress, self-care, and sexual satisfaction, plus standardized and personalized symptoms. See the Methods section in Multimedia Appendix 1 for further details on development, personas, technical notes, and symptom tracking.

**Table 1:** Overview of the Odeya intervention modules.

Module	Title	Purpose and key topics	Relational focus
1	You are not alone	Introduces sexual dysfunction and its interplay with endometriosis; fosters health literacy and support.	Normalizes relational strain within the biopsychosocial framework of sexual distress.
2	My body	Builds body awareness and sexual anatomy knowledge; covers stimulation techniques for solo and partnered activity.	Encourages exploration of bodily responses during solo and partnered touch.
3	My pain is real	Explains pain mechanisms and central sensitization; provides strategies for sexual pain management.	Promotes communication about pain, joint coping, and coregulation during intimacy.
4	My sexual self	Explores personal needs, preferences, boundaries, and the role of fantasies in sexual agency.	Encourages sharing preferences and boundaries in communication with partners.
5	My emotional network	Addresses dysfunctional beliefs using the Fear-Avoidance Model and cognitive restructuring.	Reflects on how emotional and cognitive patterns affect relationship dynamics and intimacy.
6	My sexual communication	Enhances communication skills and intimacy through couples’ exercises (eg, sensate focus).	The full module has a relationship focus. Emphasizes sexuality as relational communication and includes guided communication strategies and sensate focus exercises for couples.
7	My sexual response	Explains the sexual response cycle in a partnered context, contextual influences, and effects of stress on arousal.	The full module has a relationship focus. Addresses dyadic factors influencing arousal, responsiveness, and shared satisfaction.
8	My resources	Summarizes learnings; develops personalized routines and booster strategies for sustained sexual health.	Encourages maintaining intimacy and open communication with partners.

### Procedure

Recruitment took place from May to October 2024. Participants were recruited online through study announcements posted by endometriosis associations on their websites, Instagram accounts, and in endometriosis-related Facebook groups in Germany, Austria, and Switzerland. In-person recruitment took place via the Department of Gynecology at Charité–Universitätsmedizin Berlin and through a flyer campaign in outpatient gynecological practices across Germany (see Methods section in Multimedia Appendix 1 for selection procedures). Interested individuals emailed the study team, provided medical documentation of suspected or confirmed endometriosis or adenomyosis, and received study information. A pseudonym and masked email address were created for each participant [134] using the tool AnonAddy to protect participant identity. For screening and follow-up, they received a link to the data management platform REDCap (Research Electronic Data Capture; Vanderbilt University) [135,136] via email hosted at Charité–Universitätsmedizin Berlin. Participants were invited to a telephone interview with a clinical psychologist if further clarification was required. After randomization, the IG received access to the Odeya app. Qualitative interviews were offered to three groups: (1) IG dropouts, (2) IG completers, and (3) CG completers.

### Randomization

Balanced block randomization with 4 blocks was generated in R software (version 4.5.1; R Foundation for Statistical Computing) and implemented in REDCap (version 15.5.29) by the research team to ensure a 1:1 allocation ratio between the IG and CG. Randomization was stratified by relationship status (single vs in a relationship) and age (<30 vs ≥30 years). Participants were informed of their allocation.

### Quantitative Measures

This study assessed (1) adherence through module completion, app use duration, dropout status (IG: >4 weeks inactivity with module-locked assessments; CG: nonresponse to scheduled questionnaires), and symptom tracking activity; (2) acceptability, measured with the Client Satisfaction Questionnaire-Internet (CSQ-I), the German mHealth App Usability Questionnaire (G-MAUQ), and a single Visual Analog Scale for Client Satisfaction item on overall satisfaction; (3) safety, evaluated with the Inventory for the balanced assessment of Negative Effects of Psychotherapy-Online Intervention (INEP-ON) and self-reported changes in health status; and (4) sexual and health-related outcomes, including the FSDS-DAO, Female Sexual Function Index-German version (FSFI-d), Fear of Sexuality Questionnaire (FSQ), Vaginal Penetration Cognition Questionnaire (VPCQ), Central Sensitization Inventory-German version (CSI-GE), Partnership Questionnaire (PFB), and the Patient-Reported Outcome Measurement Information System-29-Item Profile (PROMIS-29). Detailed descriptions of assessment time points, selected measures, and additional questionnaires implemented in the broader study framework are provided in Multimedia Appendix 1 (Methods section and Table S1 in Multimedia Appendix 1).

### Statistical Analysis

Descriptive statistics (means, SDs, and medians with IQR) were computed for all sexual and health-related continuous variables at baseline (T0), T1, T2, and 6-month follow-up (T3). Change scores (Δ=value_t_ – value_0_) were analyzed with linear models for continuous outcomes. Models included group, time, and their interaction, adjusted for the respective baseline value. Adjusted mean changes and 95% CIs were obtained via the emmeans package in R software (version 4.5.1) and visualized. Adjusted between-group differences (IG-CG) are presented as mean differences with 95% CIs; *P* values were not shown, given the exploratory design. Standardized effect sizes (adjusted Cohen d; small: ≥0.2; medium: ≥0.5; large: ≥0.8) [137] were computed for the comparison of change to baseline means. For descriptive reporting, pooled SDs of the individual change scores per time point were added. As all questionnaires used mandatory fields, no item-level missing data occurred. Missingness was limited to uncompleted measurement time points and handled by available-case analysis without imputation. A predefined target sample size of n=60 was based on feasibility considerations within the mixed methods pilot design.

### Qualitative Interviews

#### Overview

The qualitative substudy included 16 participants sampled from IG dropouts (n=11; mean age 29.36, SD 3.91 years), IG completers (n=3; mean age 37.5, SD 13.44 years), and CG completers (n=2; mean 33.67, SD 5.69 years; see Table S2 in Multimedia Appendix 1 for participant characteristics). While all IG participants were invited, CG completers were recruited stepwise using criterion sampling to ensure sociodemographic diversity (age and relationship status) due to feasibility constraints. Given the small, uneven subgroup sizes, the qualitative data were used to capture a breadth of perspectives and to identify barriers and facilitators relevant to feasibility and implementation. Interviews and analysis proceeded iteratively; recruitment ended when no additional overarching feasibility-relevant issues were identified in the final interviews (further details in Methods section in Multimedia Appendix 1). Three distinct semistructured interview guides (Multimedia Appendix 3) were developed for each participant group to explore usability, engagement barriers, perceived helpfulness, and contextual factors.

#### Qualitative Data Analysis

IG dropout data were analyzed using a free-listing approach, a qualitative elicitation method that generates structured and quantifiable data by asking participants to spontaneously enumerate all responses relevant to a prompt. This method captures how individuals naturally conceptualize and prioritize health-related experiences, making it particularly useful for identifying perceived barriers, needs, and salient usability issues from participants’ own language [64,94]. Lists were subsequently cleaned, consolidated, and organized into categories through iterative coding and consensus discussions [95]. Thematic salience was derived by examining the frequency, following established guidelines [96]. Interviews with IG and CG completers were transcribed using *f4transkript* [97] and analyzed with a qualitative content analysis following Schreier’s toolbox model [98]. Coding was conducted inductively: an initial coding frame was developed from the data through pilot coding, iteratively refined as further transcripts were coded, and subsequently applied to the full dataset in MAXQDA (version 24.3; VERBI Software GmbH). Analytical rigor was supported through memo-writing and regular peer debriefing within the research team. Further details are provided in Multimedia Appendix 1 (Methods).

### Triangulation of Qualitative and Quantitative Data

In line with the expansion-type mixed methods design, quantitative and qualitative data were analyzed separately by researchers with complementary methodological expertise. Integration occurred at the interpretation stage through triangulation, whereby findings from both data strands were systematically compared and synthesized in relation to study end points through iterative discussion within the research team. Integrated findings are presented in a joint display table.

### Ethical Considerations

The human participant study was approved by the Ethics Committee of Charité–Universitätsmedizin Berlin (EA4/217/23). Participants received no compensation and could contact the research team for technical support during the study period. Data were collected and managed in REDCap hosted at Charité. Written informed consent was obtained via postal mail.

## Results

### Characteristics of Study Population

**Figure 2:**
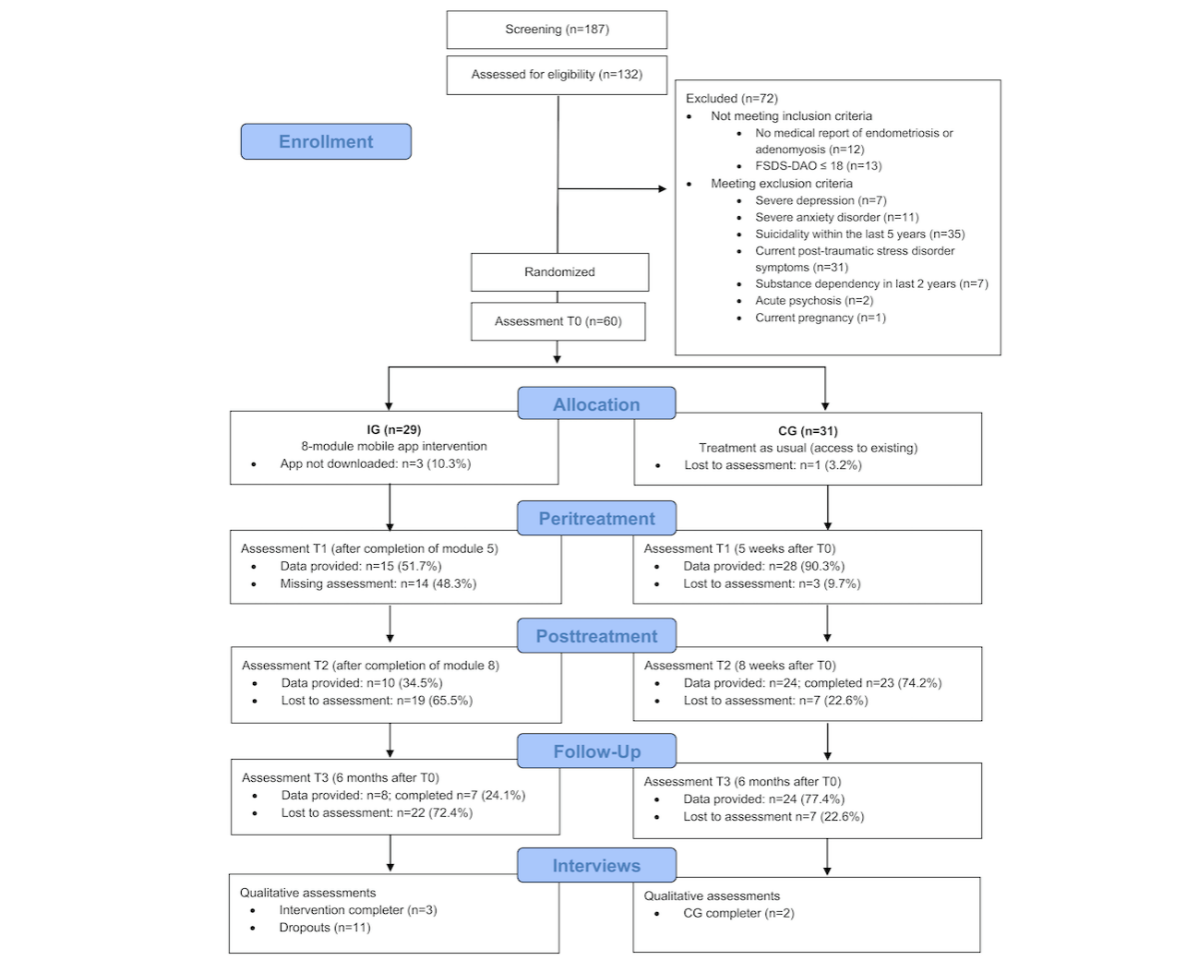
Flowchart of participants. CG: control group; FSDS-DAO: Female Sexual Distress Scale-Desire/Arousal/Orgasm; IG: intervention group.

Between July and November 2024, 187 individuals were screened. Of those, 132 completed the screening process, and 72 were excluded due to eligibility screening (Figure 2). After randomization, 29 women were assigned to the IG, and 31 were assigned to the CG. The mean age of the 60 included participants was 31.12 (SD 6.67) years, with ages ranging from 21 to 59 years (Table 2). Most participants (51/60, 85%) were in relationships and reported high relationship satisfaction (median 8.0, IQR 7.0-9.0). The overall level of education was high, with 81.7% (49/60) having 12 or more years of education. Only 1.7% (1/60) had previously accessed sex and couples therapy. However, half of the participants reported previous experience with psychotherapy. For information on motivation, participation expectations, and FSDS-DAO scores before and after randomization, see the Methods section in Multimedia Appendix 1.

**Table 2:** Characteristics of the study population at baseline (T0) stratified by intervention group (IG) and control group (CG). Percentages refer to column totals.

Characteristic			Total (N=60)		IG (n=29)		CG (n=31)
Age (years), mean (SD)			31.12 (6.67)		31.1 (5.29)		31.13 (7.83)
In a relationship, n (%)			51 (85)		26 (89.7)		25 (80.6)
Relationship duration^a^, mean (SD)			75.80 (67.64)		69.52 (56.86)		82.33 (78.01)
Relationship satisfaction (0-10)^b^, median (IQR)			8.0 (7.0-9.0)		8.0 (7.0-9.0)		8.0 (7.0-9.0)
Education ≥12 years, n (%)			49 (81.7)		23 (79.3)		26 (83.9)
Urban residence, n (%)			33 (55)		16 (55.2)		17 (54.8)
Heterosexual, n (%)			51 (85)		23 (79.3)		28 (90.3)
Partnered intimacy^c,d^, n (%)			47 (78.3)		24 (82.8)		23 (74.2)
Masturbation^c^, n (%)			20 (33.3)		7 (24.1)		13 (41.9)
Religious, n (%)			23 (38.3)		10 (34.5)		13 (41.9)
Histologically confirmed endometriosis, n (%)			52 (86.7)		26 (89.7)		26 (83.9)
Previous operation, n (%)			53 (88.3)		26 (89.7)		27 (87.1)
**Hormonal medication, n** **(%)**							
	Progestin-only contraceptive pill	17 (28.3)		10 (34.5)		7 (22.6)	
	Combined oral contraceptive pill	6 (10)		2 (6.9)		4 (12.9)	
Sex therapy or couples therapy, n (%)			1 (1.7)		0 (0)		1 (3.2)
Psychotherapy, n (%)			31 (51.7)		17 (58.6)		14 (45.2)
**Other diagnoses, n** **(%)**							
	PCOS^e^	0 (0)		0 (0)		0 (0)	
	PMS^f^	13 (21.7)		8 (27.6)		5 (16.1)	
	Uterus myomatosus	3 (5)		3 (10.3)		0 (0)	
	Uterus prolapse	1 (1.7)		0 (0)		1 (3.2)	
	Incontinence	3 (5)		1 (3.4)		2 (6.5)	
	Infertility	1 (1.7)		1 (3.4)		0 (0)	
	Vulvodynia	1 (1.7)		0 (0)		1 (3.2)	
	Lichen sclerosus	0 (0)		0 (0)		0 (0)	
	Cancer	2 (3.3)		2 (6.9)		0 (0)	
**Lifestyle, n** **(%)**							
	Physical activity	41 (68.3)		22 (75.9)		19 (61.3)	
	Healthy diet	53 (88.3)		25 (86.2)		28 (90.3)	
	Smoking	5 (8.3)		2 (6.9)		3 (9.7)	
	Alcohol consumption	8 (13.3)		3 (10.3)		5 (16.1)	
BDI-II^g^ (0-63), mean (SD)			13.3 (7.07)		13.45 (6.99)		13.16 (7.26)
GAD-7^h^ (0-21), mean (SD)			5.63 (3.4)		6.55 (3.72)		4.77 (2.88)
SSP-F^i^, n (%)			54 (90)		25 (86.2)		29 (93.5)
BSI GSI^j^, mean (SD)			54.07 (11.88)		54.86 (12.74)		53.32 (11.18)
BSI PSDI^k^, mean (SD)			53.53 (10.14)		54.52 (11.33)		52.61 (8.98)
BSI PST^l^, mean (SD)			55.05 (12.73)		55.45 (12.96)		54.68 (12.72)
CTQ^m^: Sexual Abuse^n^, n (%)			7 (11.7)		3 (10.3)		4 (12.9)
CTQ: Any Trauma^o^, n (%)			22 (36.7)		9 (31)		13 (41.9)

^a^In months.

^b^Higher values indicate better outcomes.

^c^More than once per week.

^d^Partnered intimacy referred to activities such as cuddling and kissing.

^e^PCOS: polycystic ovary syndrome.

^f^PMS: premenstrual syndrome.

^g^BDI-II: Beck-Depression-Inventory-II.

^h^GAD-7: Generalized Anxiety Disorder Scale-7.

^i^SSP-F: Screening for Sexual Problems.

^j^BSI GSI: Brief Symptom Inventory Global Severity Index.

^k^BSI PSDI: Brief Symptom Inventory Positive Symptom Distress Index.

^l^BSI PST: Brief Symptom Inventory Positive Symptom Total.

^m^CTQ: Childhood Trauma Questionnaire.

^n^Sexual trauma was defined using the Childhood Trauma Questionnaire Sexual Abuse Subscale, with a cutoff score of 8 [138].

^o^Any trauma was defined as meeting the Childhood Trauma Questionnaire cutoff for at least moderate severity [138].

### Main Findings

#### Overview

The following section reports adherence and user behavior, including module completion, dropout rates, time spent in the app, reasons for discontinuation, and use of the symptom tracker, as well as acceptance and user satisfaction assessed with validated questionnaires. Qualitative facilitators and barriers to adherence and acceptance follow in subsequent sections. Safety outcomes comprised balanced effects, self-reported health changes, and stressful life events.

#### Adherence and User Behavior

##### Dropout Rates, Time, and Reasons

Participants in the IG completed a median of 6 (IQR 2-8) modules (mean 4.9, SD 2.97; 4.9/8, 61.2% of the total content). All 8 modules were completed by 34.5% (10/29) of the IG, with a dropout rate of 65.5% (19/29), which is higher than in the CG (7/31, 22.6%). Mean app usage duration, defined as the time from first log-in until either completion of module 8 or the last recorded in-app activity (therapeutic content or symptom tracking), was 15 weeks (range 0-30) in the IG. Among completers, the mean duration was 18 (range 10-30) weeks, whereas noncompleters used the app for an average of 13 (range 0-30) weeks. IG dropouts were distributed throughout the course of the intervention: before starting the app (n=3), after module 1 (n=3), module 2 (n=2), module 3 (n=1), module 4 (n=4), module 5 (n=1), module 6 (n=4), and module 7 (n=1). Baseline characteristics (Table S3) and baseline values of outcomes (Table S4) of IG completers and IG dropouts are presented in Multimedia Appendix 1. Health care behaviors showed little change; only isolated therapy initiations occurred (Results section in Multimedia Appendix 1). Reasons for dropout included time constraints, technical difficulties, life changes, and perceived length of app units (Tables S5-S7 in Multimedia Appendix 1).

##### Adherence Facilitators and Barriers

High initial motivation and the persona-based progress stories supported adherence:

You also got to witness the progress of the three women, and that was motivating and encouraging.

IG1

Several participants felt acknowledged by the intervention, contrasting it with previous clinical encounters where their concerns had not been taken seriously.

Participants identified unexpectedly high time demands and emotional strain of prolonged self-reflection as key barriers:

It kept getting longer and more extensive.... I think it was supposed to be 50 minutes per module..., but I sometimes needed almost three times as long.... It was really exhausting to constantly engage with myself.

IG1

Some wished for more interaction with health care professionals or peers and reported difficulties applying insights to daily life and relationships.

When it comes to communication in the relationship that was also a very big topic in the app and I’ve always struggled with that. There were helpful impulses, but I couldn’t really implement them at that point.

IG2

Technical issues—videos stopping without replay options, restrictive response formats, and static text fields limiting review of longer entries—further affected usability and adherence.

##### Use of Symptom Tracker: Frequency and Evaluation

**Figure 3:**
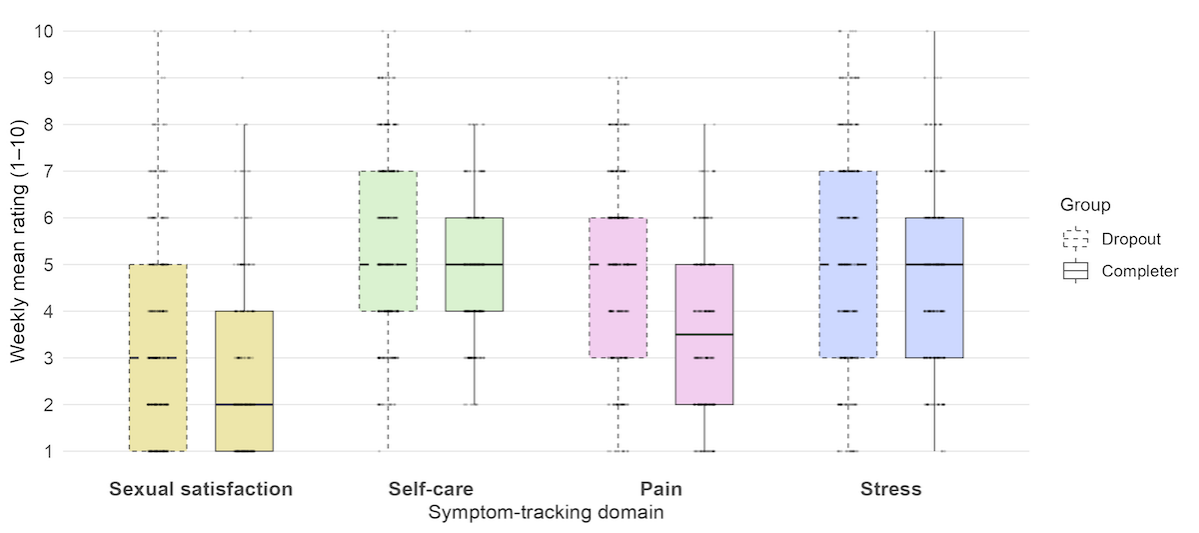
Rating of intensity levels of sexual satisfaction, self-care, pain, and stress in the IG, stratified for completers (n=10) and dropouts (n=15), showing median rating averaged across the usage weeks.

Symptom tracking usage varied widely across participants. The average number of tracking entries during intervention was 18.34 (SD 23.77; range 0-99), with completers averaging 23.20 (SD 34.03; range 4-99) and dropouts 15.79 (SD 16.73; range 0-46). The most frequently self-selected tracked symptoms included, for emotional states: feeling relaxed, sad, or tired; for physical symptoms: bowel problems, intake of pain medication or hormones, and bladder problems; for social aspects: participation in social activities, sports, or work-related stress; and for sexuality: no sexual activity, masturbation, or sexual intercourse. Figure 3 shows the mean tracked levels of sexual satisfaction, self-care, pain, and stress.

Qualitative feedback on tracking reminders was mixed; some participants perceived them as redundant or burdensome, while one reported them as helpful.

#### Acceptance

##### User Satisfaction

At midintervention, the median satisfaction score was 8.00 (IQR 7.0-8.0; mean 7.53, SD 1.36; n=15), and at postintervention (T2), 7.50 (IQR 6.0-8.0; mean 7.00, SD 1.83; n=10) on a 10-point scale (Visual Analog Scale for Client Satisfaction). At T2, CSQ-I ratings had a mean of 26.60 (SD 4.12) of 32. The CSQ-I sum of items assessing general satisfaction (items 1, 2, 3, 5, and 7) averaged 16.30 (SD 2.45), item 4 assessing recommendation 3.50 (SD 0.71), item 6 assessing helpfulness 3.10 (SD 0.88), and item 8 assessing likelihood of reuse 3.70 (SD 0.67). Based on these ratings, 80% (8/10) of participants agreed or strongly agreed with CSQ-I total items, 90% (9/10) reported that they would recommend or reuse the intervention, and 70% (7/10) reported that the intervention was helpful and fulfilled their general satisfaction. Usability, assessed with the G-MAUQ, showed a total score of 5.38 (SD 0.74), with subscales of ease of use (mean 6.46, SD 0.64), interface satisfaction (mean 5.28, SD 0.78), and usefulness (mean 4.46, SD 1.69). For detailed ratings, including median, minimum, and maximum values, see Table S8 in Multimedia Appendix 1.

These findings were closely mirrored in participant interviews, where usability, app design, and comprehensive content were highlighted as facilitating factors.

First of all, in terms of the technical aspects and structure, I found the app very clear and easy to use.

IG1

Qualitatively, some users expressed discomfort or ambivalence about specific exercises, such as guided masturbation or vulva self-exploration.

##### Facilitators and Barriers to Acceptance

Multimedia elements and the option to proceed at one’s own pace were highly valued. Participants appreciated the intervention’s diverse components, including normalization through personas (“not feeling alone”), psychoeducation, practical and communication exercises, structured reflection, partners’ involvement, and gradual exposure to sensitive topics, especially the sensate focus exercises.

However, several barriers were identified. Technical issues and content overload inhibited satisfaction for some. These frustrations might explain lower satisfaction scores or disengagement in some cases. Weekly exercises were challenging, leading to self-doubt and frustration, although these feelings were partly mitigated by greater awareness of social influences, as one participant described:

Sometimes I felt very frustrated, doubting if it was my fault.... I even felt guilty after reading or doing certain exercises.... However, I was able to balance this by reflecting on what I’d learned from my parents’ attitudes toward sexuality...phrases from friends or relationships. These insights gave me “aha” moments, helping me feel less desperate and understand that other factors might be involved.

IG3

#### Safety

##### Balanced Effects

The most frequently endorsed positive effects in the INEP-ON outcome were the helpfulness of new ways of thinking (T2: 10/10, 100%; T3: 6/7, 85.7%) and moderator support (T2: 9/10, 90%; T3: 4/6, 57.1%; Table S9 in Multimedia Appendix 1). Increased motivation for psychotherapy was reported by 60% (6/10) at T2 and 42.9% (3/7) at T3. Improved overall well-being was noted by 90% (9/10) postintervention, but only 28.6% (2/7) at follow-up, while 57.1% (4/7) reported deterioration. Negative responses on balanced items were otherwise rare (≤10% at T2 and <30% at T3; see Results section in Multimedia Appendix 1). Items addressing exclusively negative effects (Table S10 in Multimedia Appendix 1) showed longer periods of not feeling well in 50% (5/10) at T2 and 71.4% (5/7) at T3 (see Results section in Multimedia Appendix 1 for details).

##### Self-Reported Health Changes and Stressful Life Events

A total of 6 women in the IG and 8 in the CG reported events, including new medical diagnoses (eg, cardiac arrhythmia, asthma, adenomyosis, suspected lipedema, and migraine), hospitalizations, bereavement, or relationship breakups. In the IG, only 1 affected participant dropped out; the others completed the study.

### Secondary Outcomes

#### Overview

**Figure 4:**
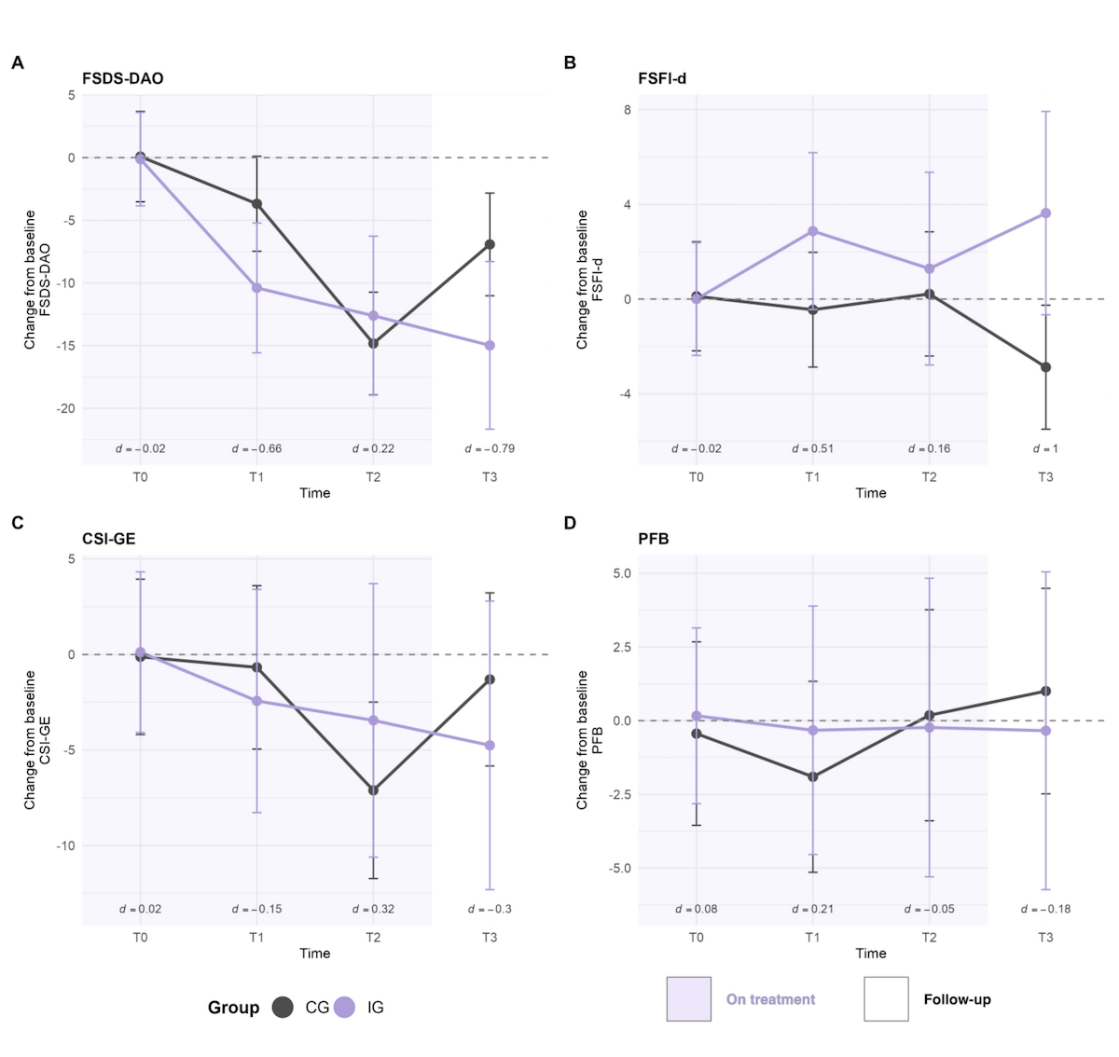
Sexual health outcomes over time as changes from baseline (Δ) for intervention group (IG) and control group (CG) across T0-T3. Points and lines depict estimated marginal means of the change scores; error bars indicate 95% CIs. Above the x-axis, the between-group effect size (d) from the linear model (Δ ~ Group × Time + Baseline) is shown for each time point. Shaded backgrounds denote study phases (on treatment: T0-T2; follow-up: T3). Panels A-D correspond to outcomes. CSI-GE: Central Sensitization Inventory-German version; FSDS-DAO: Female Sexual Distress Scale-Desire/Arousal/Orgasm; FSFI-d: Female Sexual Function Index-German version; PFB: Partnership Questionnaire; T0: baseline; T1: after module 5/5 weeks after baseline; T2: after module 8/8 weeks after baseline; T3: 6-month follow-up after baseline.

Secondary outcomes included exploratory analyses of changes in sexual health (FSDS-DAO, FSFI-d, FSQ, VPCQ, CSI-GE), relationship (PFB), and overall health (PROMIS-29). Table S11 in Multimedia Appendix 1 reports mean scores and Table S12 baseline-adjusted changes with effect sizes for all outcomes. Figure 4 illustrates sexual health outcomes based on total scores.

#### Sexual Health and Relationship-Related Outcomes

The IG showed a stronger early reduction in sexual distress (FSDS-DAO) (T1: IG Δ=–10.39 vs CG Δ=–3.68; between groups Δ=–6.71, 95% CI –13.13 to –0.29; *d*=–0.66) while both groups improved by T2 (Figure 4 and Table S12 in Multimedia Appendix 1). At T3 the IG again showed a stronger reduction (T3: Δ=–8.05, 95% CI –15.89 to –0.22; *d*=–0.79).

Sexual function (FSFI-d) improved continuously in the IG (T1-T3 Δ=1.29-3.63), with the largest between-group difference at T3 (Δ=6.51, 95% CI 1.48-11.55; *d*=1.00).

Fear of coitus (FSQ) showed small between-group effects, with the IG showing greater improvement at T1 (*d*=−0.21) and T3 (*d*=−0.14), but the CG at T2 (*d*=0.17). Fear of noncoital activity decreased continuously in the IG by about 0.6 points at T2 (scale ranging from 5-25), with small to large effects between groups (*d*=−0.14 to −0.84; Table S10 in Multimedia Appendix 1).

Regarding vaginal penetration cognition (VPCQ), results were mixed, with small to moderate improvements in the IG, primarily at T1 and T3 (eg, between-group effects: at T3, control cognitions d=0.48; catastrophic and pain cognitions d=−0.17). The only scale showing continuous improvement favoring the IG was incompatibility cognitions, with between-group effect sizes ranging from d=−0.34 to −0.89.

Central sensitization (CSI-GE) improved in both groups. Small effects favored the IG at T1 (*d*=−0.15) and T3 (*d*=−0.30), while at T2 a small effect favored the CG (*d*=0.32).

Partnership quality (PFB) demonstrated only small changes over time (T1: Δ=1.58; d=0.21; T2: Δ=−0.42; d=−0.05; T3: Δ=−1.34; d=−0.18). Subscale effects were likewise small: disruptive behavior worsened slightly in the IG at T3 (d=0.35), whereas communication improved in the IG at T1 (d=0.26) and T3 (d=0.21), with no change at T2 (d=−0.01).

Qualitative interviews indicated that IG participants frequently reported new positive sexual experiences:

Using the app helped me to rediscover how to have positive experiences with sexuality—across the entire spectrum of what sexuality can be.

IG1

Several reported that their perception and communication of sexual pain improved, although complete pain relief was rarely achieved:

From the communication perspective, I felt that I was often able to say, “Okay, I have pain now,” and I could localize it, to give him that feedback.

IG3

Further, IG participants described gaining more courage and reduced fear and avoidance of sexual activities, reporting increased confidence in approaching both coital and noncoital intimacy:

To find the courage to start again and actually do something. And also, those small steps—that was exactly what I needed. I can imagine many others feel the same, because there’s always that fear of penetrative sex.

IG1

Qualitative analyses revealed that participants in the IG experienced increased openness and more frequent communication with their partners regarding sexuality and recognized the issue as a shared concern affecting both partners, rather than an individual problem. One participant noted:

In the beginning, when I started using the app, I really tried to communicate more with my partner.... It’s usually such an uncomfortable topic for me—I’ve never talked about it openly. But I tried to tell him about the app, showed him parts of it.... I realized, it’s not just my issue, it’s something that affects the relationship too, and we’re both suffering in some way.

IG3

Additionally, several participants described a shift toward a more constructive and collaborative dynamic, moving away from blame:

We talked about that very openly—that it’s not about anyone being at fault or him hurting me or anything like that, but rather that we go through the process together and figure out, together, what feels good and what doesn’t.

IG3

Although CG participants had no access, qualitative interviews suggested that study participation itself fostered self-reflection prompted by study questionnaires.

I actually liked that it forced me to keep reflecting...on what’s going on in that part of my life, my sexuality or lack of libido. [It was really] helpful].

CG1

CG participants also reported positively impacting life events over the course of the study (eg, hormonal changes and changes in sexual life circumstances) and expressed continued interest in the intervention after the study.

#### Health-Related Quality of Life

Results for the PROMIS-29 domains were mixed overall. Anxiety, depression, fatigue, and physical function improved in both groups at T2, although between-group comparisons favored the CG at several time points. In contrast, social participation, pain interference, and pain intensity improved across all time points in the IG, with between-group effects favoring the IG at T2 and T3 and showing moderate to large effect sizes.

Qualitatively, participants in the IG attributed enhanced emotional regulation and reduced distress to the intervention, linking these psychological changes to better physical health management:

That was one of my goals—to somehow get out of this negative emotional state. And it actually worked. Of course, there are still phases or moments when you think, “Hmm...” But then things come to mind—things you can do, right? And you realize, okay, even if it’s not working right now, that’s okay too—it’s not the end of the world.

IG1

Self-reflection and body awareness were recurring qualitative themes reported by both groups, further elucidating why both showed improvement in those domains.

### Integration of Quantitative and Qualitative Findings

The integration of quantitative outcomes and qualitative interviews (Table 3) provides a nuanced picture of the intervention’s effects on sexual health, relationships, well-being, and user engagement. Quantitative findings contextualized quantitative improvements and highlighted adherence challenges and the complexity of symptom management.

**Table 3:** Synthesis: integration of quantitative and qualitative results. Intervention group (IG) > control group (CG) indicates between-group differences in favor of the intervention group, whereas IG < CG indicates differences in favor of the control group.

Domain	Quantitative findings	Qualitative themes	Synthesis
Adherence	IG completion 34.5% (10/29), dropout 65.5% (19/29) vs CG 22.6% (7/31) Symptom tracking more frequent in completers Frequent use of individual symptom feature Outside app: start of psychotherapy, no change in costs	Facilitator: persona stories Barriers: emotional strain, time demands Reminders: helpful or confusing Dropout: time, technical issues, life changes Need: more professional/patient interaction	High dropout aligns with time/emotional strain; progress stories may support adherence. Mixed feedback on tracking suggests the need for customizable reminders. Behavior outside app indicates broader health-seeking changes.
Acceptance	CSQ-I^a^ mean 26.6 (SD 4.1) of 32 G-MAUQ^b^ mean 5.4 (SD 0.7) of 7; “Ease of use” mean 6.5 (SD 0.6) of 7 Mean price €83 (range €0-€300; €1=US $1.17); 30% (3/10) no self-pay	Facilitators: clarity, multimedia, flexibility, reimbursement Barriers: technical issues, content overload	High satisfaction matches positive reports; barriers may explain disengagement. Payment variability underscores the need for reimbursement.
Safety	INEP-ON^c^: learned strategies, moderator support “Negative events” rare (≤10% in T2, <30% in T3); mainly periods of not feeling well	Reports of health changes, stressful life events in both groups	Findings suggest overall safety; negative responses linked more to external stressors than intervention.
Sexual health	FSDS-DAO^d^: improved in both groups, IG > CG at T1, T3 FSFI-d^e^: IG > CG FSQ^f^ “coital”: improved in both, IG vs CG mixed; “noncoital”: IG > CG VPCQ^g^ “control,” “catastrophic”: improved in both groups, IG vs CG mixed; “Incompatibility”: IG > CG PFB^h^: no change at T2; IG vs CG mixed; “Communication”: IG > CG at T1, T3 CSI-GE^i^: improved in both groups, IG > CG at T1; T3, IG < CG at T2	Facilitators: rediscovery of positive experiences, improved pain communication, openness, collaboration Barriers: ambivalence toward some exercises CG: self-reflection via questionnaires and self-applied tools/books	Quantitative gains align with reports of reduced distress and better sex-related communication. Although some changes were not strongly reflected in quantitative scores, participants described meaningful qualitative improvements. CG improvements may be attributed to study participation and self-reflection.
Overall Health	PROMIS-29^j^: “anxiety,” “depression,” “fatigue,” “physical function” improved at T2 in both groups, mostly IG < CG; “Social participation”: IG > CG at T3; “Pain interference”, “pain intensity”: IG > CG at T2, T3.	IG: improved emotional regulation, distress reduction Both: more self-reflection, body awareness	Both groups, especially the CG, showed broad improvements in psychological well-being and physical functioning, while the IG exhibited reductions in pain interference and intensity. Qualitative reports of greater emotional regulation, open partner communication, and body awareness in the IG support these findings, indicating that self-regulatory and relational processes may reduce pain-related avoidance and attentional capture by pain.

^a^CSQ-I: Client Satisfaction Questionnaire-Internet Version.

^b^G-MAUQ: German mHealth App Usability Questionnaire.

^c^INEP-ON: Inventory for the balanced assessment of Negative Effects of Psychotherapy.

^d^FSDS-DAO: Female Sexual Distress Scale-Desire/Arousal/Orgasm.

^e^FSFI-d: Female Sexual Function Index-German version.

^f^FSQ: Fear of Sexuality Questionnaire.

^g^VPCQ: Vaginal Penetration Cognition Questionnaire.

^h^PFB: Partnership Questionnaire.

^i^CSI-GE: Central Sensitization Inventory-German Version.

^j^PROMIS-29: Patient-Reported Outcome Measurement Information System-29-Item Profile.

## Discussion

### Principal Findings

This pilot implementation study examined the adherence, acceptability, safety, and exploratory effects of the self-guided Odeya app for women with sexual distress and diagnosed or suspected endometriosis. Adherence was moderate, with most completers working through several modules over an average duration of 18 weeks, while dropout was high. Overall satisfaction with the app was strong, and no negative life and health changes were attributed to the intervention. Quantitative outcomes showed reductions in sexual distress in both groups, with some advantages for the IG in sexual function, penetration-related fears, and partner communication. Broader health and mental health outcomes showed mixed but generally positive changes across groups. Qualitative feedback supported these trends, describing more positive sexual experiences, improved partner communication, greater pain awareness, and reduced fear of penetration.

### Adherence

#### Overview

The intervention dropout rates in our study were notably high, a phenomenon typically reported in digital interventions for chronic conditions. A meta-analysis found a pooled dropout rate of 43% across app-based interventions for chronic diseases [76]. In the HelloBetter Vaginismus DiGA effectiveness trial, Zarski et al [50] reported dropout of 22% postintervention among women with dyspareunia, with participants completing on average 6 of 8 modules. In an early study, the Endo App retained 64.4% (29/45) of endometriosis patients in week 12 [61]. The German DiGA registry (BfArM) reports low attrition in IGs for several DiGAs, including the Endo App (3.75% dropout; NCT04883073) [60], Kranus Edera for erectile dysfunction (4.1%) [59], and Kranus Lutera for incontinence (5.4%) [62]. In comparison with these trials, dropout in our study was higher in the IG than in the CG [50,59,61,62]. Several factors may explain the comparatively high dropout in our sample.

#### Health Status

First, our study population comprised individuals with endometriosis and sexual distress, a particularly complex clinical profile that may require more intensive support than fully self-guided interventions can provide [69]. Unlike trials with stricter exclusion criteria (eg, excluding chronic pain or moderate depression [49,50,58], recent medication or surgery changes [60], chronic infections [62], or postsurgical/cardiovascular risk) [59], our study was deliberately inclusive, excluding only severe depressive or anxiety symptoms. This approach enhanced external validity but may have increased attrition among participants facing multiple health burdens. At baseline, dropouts reported lower quality of life and greater symptom burden (anxiety, depression, stress, fatigue, and pain) yet less sexual distress and better sexual function than completers. Visual inspection of the symptom-tracking data suggested slightly higher median values among dropouts for sexual satisfaction and pain compared to IG completers. This health status pattern may also explain the continuous dropout observed in our study, as disengagement likely reflects participants’ health burden rather than low initial motivation or lack of interest in the intervention—contrasting with the early-stage dropout typically reported in digital mental health apps [139,140]. Some studies found no link between depression or pain and attrition [79,81]. However, a systematic review and a meta-analysis identified baseline depression and comorbid anxiety as predictors of dropout [77,80]. Further, most participants were in relationships. Being partnered has been identified as a risk factor for sexual dysfunction with distress [12], whereas being single has already been associated with higher dropout [81].

#### App Engagement

Second, app engagement and adherence may operate in both directions. While active tracking could facilitate greater adherence, it is also plausible that individuals with better health or mental well-being were more likely to engage with the app. Dropouts tracked symptoms less frequently, indicating lower initial engagement with self-monitoring features. Early engagement predicts adherence in digital health programs [78,81,82] and may mark a critical window for retention strategies. Evidence on the link between tracking and adherence in digital health interventions is mixed: some studies report benefits of consistent symptom tracking, while others show only transient effects [68,86,89]. In our study, reduced tracking and higher dropout among participants with elevated anxiety and depression suggest that mental health symptoms hinder sustained engagement. This aligns with meta-analytic evidence showing that engagement modestly predicts improved outcomes, with specific indicators such as module completion being most predictive [83].

#### Program Design

Third, compensation structures and intervention duration may have influenced adherence. Studies with better adherence rates often provided participant compensation or had shorter intervention periods [84], factors that can significantly impact engagement and completion rates. Although a 12-week duration is clinically appropriate and common [50,58,59,61,62], it may be demanding for participants with chronic pain and its psychological burden, as noted in prior studies [141]; furthermore, modules were reported to exceed the displayed module duration of 60 minutes.

### Acceptance and User Experience

#### Overview

Quantitative results showed high user satisfaction, aligning with findings in women with genito-pelvic pain/penetration disorder (CSQ-I mean 28.03, SD 3.96; 67/72, 93% satisfaction) [50] and supported by qualitative reports of positive experiences. Similar satisfaction levels have been reported in other digital interventions across different patient populations using the CSQ-I [65,72]. Usability (G-MAUQ) ratings were particularly high for ease of use, likely reflecting the patient-centered development process (E Kosman, MSc, et al, unpublished data, 2026) and the app’s reduced functions, with scores comparable to other digital interventions [66,85]. Perceived usefulness was slightly lower in our study, which may have contributed to attrition.

#### Content Overload and Usability as Barriers

Content overload, reflected in the high time demands of the modules and the emotional strain of sustained self-reflection [142], highlighted the challenge of balancing comprehensive content with practical feasibility for participants. Similar findings show that time-intensive digital interventions hinder engagement in this population [74]. Moreover, even seemingly minor usability issues can significantly affect user experience and sustained engagement [73,75,87].

#### Guidance

Interview participants frequently requested more clinician and peer contact—consistent with evidence that human support drives engagement [88]—and reported difficulties applying content and feeling insufficiently guided. This underscores the potential value of integrating human support into digital tools for complex chronic conditions. Interventions including personal guidance achieved better adherence [56,76] compared with our fully self-directed approach. Blended care models may enhance both engagement and acceptance by providing structured support alongside digital self-management [143].

#### Willingness to Pay

Despite high user satisfaction, willingness to pay for a comparable DiGA was limited. Participants expressed generally low willingness to pay, substantially lower than prices of comparable DiGAs in Germany [57,63], and many felt that such tools should be financed by the health care system rather than by users themselves. Similar patterns have been reported, linking reluctance to pay to expectations of public coverage, skepticism toward digital tools, and limited awareness of benefits [144].

### Safety

None of the health and life changes was attributable to the intervention. Comparisons with other digital interventions are limited, as adverse events are rarely assessed or reported in German DiGAs [71]. Our findings accord with a recent meta-analysis indicating that mental health apps rarely cause harm and do not increase adverse events versus controls [70].

### Changes in Sexual and Overall Health

Although not powered for clinical end points, the trial provided exploratory indications of improvements, with small to large effects based on between-group mean differences. Our findings are consistent with evidence that both face-to-face and online interventions can reduce sexual distress and improve sexual function and satisfaction [16,41,44], that psychoeducation increases knowledge and reduces performance anxiety [31], and that mindfulness improves desire, arousal, and satisfaction while mitigating sexual distress [45,46]. An online self-help trial for women with dyspareunia reported medium to large improvements in genital pain and penetration-related cognitions and small to medium improvements in sexual function, anxiety, and well-being [50]. A psychoeducational web-based program for cancer survivors improved sexual communication but showed limited impact on relationship outcomes [67]. Consistent with this pattern [50,51,53,54], our trial also did not affect relationship satisfaction. Qualitative data aligned with established mechanisms [16,55]: participants described sensate focus and self-stimulation exercises as helpful for improving desire, and cognitive restructuring for reducing maladaptive sexual beliefs. These processes were reflected quantitatively by reduced incompatibility cognitions in the IG. However, it should be noted that some between-group differences may reflect random error rather than genuine effects.

Consistent with prior research [50], control participants also improved in health (eg, depression, anxiety, and fatigue) and sexual distress. However, at follow-up, reductions in sexual distress persisted strongly only in the IG. Qualitative data suggest that repeated self-assessment may have acted as a catalyst for self-reflection and proactive coping, a Hawthorne-like effect [145]. Additional mechanisms include mere-measurement effects [146], hope and expectancy [147], natural recovery [50,148], and enhanced self-monitoring [149]. Despite these effects, CG participants continued to desire intervention access, indicating ongoing unmet needs.

### Implications for Future Digital Interventions

Despite the high dropout rate, satisfaction metrics in the IG indicated that engaged participants found the program helpful. Thus, content appears valuable, whereas delivery and support structures may require optimization. Following qualitative feedback indicating a desire for more guidance and professional contact, we propose key design considerations for future digital interventions (Textbox 1).

**Key design considerations to enhance engagement, adherence, and user experience in future digital sexual health interventions targeting individuals with endometriosis**

1. Hybrid approaches: incorporating human support elements—and, where appropriate, AI-driven assistance—such as periodic check-ins with health care providers, trained coaches, or moderated peer communities, may enhance engagement, adherence, and outcomes [150-152].
2. Tailored content delivery: reducing the time burden and emotional intensity of modules while maintaining therapeutic effectiveness could improve completion rates. Flexible options—such as individually selectable modules, exercises, or notifications and supplementary materials (eg, workbooks)—may better address diverse user needs [153,154].
3. Technical optimization: addressing usability issues and ensuring a seamless user experience—potentially through the integration of gamification features—is fundamental to sustaining user engagement [155].
4. Screening for intervention readiness: given the relationship between baseline psychological distress and dropout, screening for intervention readiness and providing additional support for participants at high risk of dropout may improve outcomes [156].
5. Patient involvement: beyond developing the intervention through a patient-centered approach, we recommend involving patients or patient influencers in the go-to-market phase to ensure effective reach and trust [64,157-160].

### Integrating the Relational Dimension

Beyond the structural and delivery-related design considerations outlined above, the relational dimension constitutes a further critical aspect of future digital sexual health interventions. Our findings address a recently identified structural limitation in current DiGAs for sexual dysfunctions: the insufficient consideration of the relationship dimension. While existing DiGAs primarily address individual functional parameters and measure success through standardized scores such as the International Index of Erectile Function‐5, sexual dysfunctions are frequently embedded in relational components that require dyadic intervention approaches [91].

### Future Research

Future research should test efficacy for core sexual health outcomes, particularly sexual distress, in adequately powered randomized controlled trials and, in larger samples, identify subgroups most likely to benefit. Incorporating participants’ recommendations—such as integrating the program into blended-care models with chat support or video consultations [12]—may enhance value and warrant evaluation. Longer, more flexible trials should also examine motivational drivers and dropout trajectories, as a 4-week inactivity threshold may be overly restrictive (eg, for women recovering from endometriosis surgery). Moreover, future iterations could include both optional and mandatory partner components addressing partners directly (eg, psychoeducation modules on endometriosis and its relational impact).

### Limitations

This study has several limitations. First, several methodological aspects limit the interpretability of findings. The trial was not powered to detect efficacy or between-group differences, constraining comparative analyses. Additionally, although aspects of preuse acceptability were addressed during development, they were not systematically assessed in this study. Without a dedicated prepilot acceptability study, some usability issues (eg, module length and navigation) may not have been identified beforehand. Finally, combining assessments of acceptability and preliminary effectiveness within a single early-phase trial may have constrained sampling strategies and methodological specificity. Second, dropout rates were high—particularly in the IG—resulting in substantially reduced sample sizes for both quantitative and qualitative components. This further restricts within-group analyses and impedes meaningful comparisons between the IG and CG. Participants who discontinued showed higher baseline symptom severity (eg, anxiety and depression), potentially biasing findings toward more favorable outcomes. Third, satisfaction outcomes may be influenced by recruitment and measurement constraints. Because the recruitment strategy relied primarily on online channels, the sample may overrepresent women with higher digital literacy or a stronger preference for online support formats. Satisfaction was also only assessed among women who completed the intervention due to predefined measurement timing, likely inflating acceptability ratings. Fourth, sample characteristics and condition-specific focus limit generalizability. The sample covered a broad age range (up to 59 years), representing heterogeneous life stages (eg, perimenopause and menopause) with potentially distinct biopsychosocial profiles. Participants were also highly educated and predominantly partnered, which is not representative of the wider population. Moreover, although the intervention was developed through a patient-centered approach with women affected by different gynecological conditions (E Kosman, MSc, et al, unpublished data, 2026), it was only tested in women with endometriosis or adenomyosis, further limiting generalizability. Fifth, while the intervention included optional partner-focused components, systematic involvement of partners was limited, and dyadic processes were not comprehensively addressed. This represents a conceptual limitation, given that sexual health in the context of chronic conditions such as endometriosis is inherently relational. Finally, a coding error prevented proper attribution of INEP-ON items, obscuring whether reported positive or negative changes were related to life circumstances or the intervention itself.

### Conclusions

The digital intervention Odeya appeared acceptable and safe for women who engaged with the app, with initial indications of improvements in sexual health–related outcomes among completers. Sustained engagement in fully self-guided formats, however, appears to vary by baseline psychosocial status, with individuals reporting better psychosocial health showing higher adherence. Adequately powered trials should establish efficacy, identify moderators of benefit, and determine the support intensity needed to maximize impact. Self-guided digital interventions may present one accessible and scalable component within stepped-care models for sexual health, although some users are likely to require additional guidance and personalization.

## Appendix

Multimedia Appendix 1 Supplementary methods, results, and tables.

Multimedia Appendix 2 CONSORT‐eHEALTH checklist (V 1.6.1).

Multimedia Appendix 3 Interview protocols.

## Abbreviations

BfArM: German Federal Institute for Drugs and Medical Devices (Bundesinstitut für Arzneimittel und Medizinprodukte)
CG: control group
CSI-GE: Central Sensitization Inventory-German Version
CSQ-I: Client Satisfaction Questionnaire- Internet Version
DiGA: digital health application
DSM-IV: Diagnostic and Statistical Manual of Mental Disorders, Fourth Edition
FSDS-DAO: Female Sexual Distress Scale-Desire/Arousal/Orgasm
FSFI-d: Female Sexual Function Index-German version
FSQ: Fear of Sexuality Questionnaire
G-MAUQ: German mHealth App Usability Questionnaire
ICD-11: International Classification of Diseases, 11th Revision
IG: intervention group
INEP-ON: Inventory for the balanced assessment of Negative Effects of Psychotherapy
PFB: Partnership Questionnaire
PROMIS-29: Patient-Reported Outcome Measurement Information System-29-Item Profile
PTSD: posttraumatic stress disorder
REDCap: Research Electronic Data Capture
T0: baseline
T1: after module 5 in intervention group/5 weeks after baseline in control group
T2: after module 8 in intervention group/8 weeks after baseline in control group
T3: 6-month follow-up after baseline
VPCQ: Vaginal Penetration Cognition Questionnaire

## Data Availability

The datasets analyzed during this study are not publicly available due to restrictions imposed by the ethics approval.
