# Supplementary material for: Adherence, Acceptability, and Sexual Health Outcomes of the Odeya App–Based Intervention for Sexual Distress in Women With Endometriosis: Randomized Controlled Mixed Methods Trial
Source: J Med Internet Res. 2026 Feb 19;28:e86042. doi: 10.2196/86042 (PMC12963981; doi:10.2196/86042)
Supplement: Multimedia Appendix 1 [file jmir_v28i1e86042_app1.pdf]

Multimedia Appendix 1

Corresponding Author:
Dr. med. Laura Hatzler
Institute of Sexology and Sexual Medicine
Department of Urology
Charité-Universitätsmedizin Berlin
Charité Platz 1, 10117 Berlin, Germany
Phone: +49 30 450 617 139
Fax: +49 30 529 992
E-Mail: laura.hatzler@charite.de

Methods. The additional method section details pre-study development procedures, recruitment, quantitative measures, qualitative sampling, interview procedures, and analysis.

Results. The additional result section reports motivation and expectations, FSDS-DAO scores at screening and baseline, user behavior outside the app, willingness to pay, and safety outcomes.

Table S1. Schedule of assessments and quantitative outcome measures by assessment timepoint.

Table S2. Characteristics of Qualitative Assessment Participants (*N*=16) at Baseline (T0).

Table S3. Characteristics of Intervention Completers and Dropouts at Baseline (T0).

Table S4. Screening and Baseline Values of Outcomes Stratified for IG Completers and Dropouts.

Table S5. Dropout Interview Question 1.

Table S6. Dropout Interview Question 2.

Table S7. Dropout Interview Question 3.

Table S8. Descriptive Statistics for Acceptance of the intervention at T2 (*n*=10).

Table S9. INEP-ON Balanced Changes.

Table S10. INEP-ON Negative Changes.

Table S11. Mean Scores and Standard Deviation at Baseline (T0), Mid-Intervention (T1), Post-Intervention (T2), and 3-Month-Post Intervention (T3), Stratified by Group.

Table S12. Baseline-Adjusted Mean Changes and Between-Group Differences (Linear Model).

Methods

Pre-Study Procedures

This study was preceded by a patient-centered, iterative development phase comprising: (1) need identification and validation via free-listing interviews, (2) usability testing using a “thinking aloud” protocol, and (3) content validation through focus groups, involving women with gynecological conditions (eg, endometriosis, vulvodynia, breast cancer) and sexual medicine expert.[1]

Design Rationale

Mixed-methods designs are recommended in early-phase intervention research because they integrate feasibility, acceptability, and preliminary change signals with in-depth insights into user experiences and contextual factors not captured quantitatively.[2] Integrating both strands further supports the identification of unanticipated phenomena and deepens understanding of participants’ experiences.[3]

Intervention Development, Personas, Technical Notes, and Symptom Tracking

Content was developed by an interdisciplinary gynecology–psychology team, drawing on evidence-based components from cognitive behavioral therapy,[4, 5] sex therapy,[6] and mindfulness-based cognitive therapy.[7] The structure and therapeutic rationale were guided by the circular sexual response model by Basson,[8, 9] emphasizing biopsychosocial influences on sexual function and distress. Individual modules corresponded to specific stages of this model, addressing physiological, cognitive, emotional, and relational dimensions of female sexual response.

To enhance relatability, three fictional characters – Kim (23 years), Naomi (34), and Claudia (52) – portray diverse experiences through video, audio, and text, based on clinical expertise and interviews.[1]

A technical issue during the trial temporarily prevented progression due to a text input limit; this was resolved via a software update. The therapeutic content remained unchanged throughout the intervention.

Graphs within the app visualized progress over time. Symptom selection was guided by literature[10-12] and user interviews.[1] Participants were encouraged to track daily, preferably in the evening, with reminders sent after 48 hours of inactivity.

In-Person Recruitment Procedures

Practices were identified via online searches and listings of clinics providing endometriosis care and were contacted beforehand to confirm their willingness to display study flyers. All recruitment materials included the study email address and a QR code linking directly to the study team.

Quantitative Measures

Demographics

Sociodemographic variables included age, education, income, migration background, urban/rural residence, and religious affiliation, as well as relationship status, sexual orientation, and sexual behavior. Medical variables covered general physical symptoms, pain, previous therapies, surgical procedures, medication use, menstrual cycle, and pregnancy history, with particular focus on endometriosis-related treatments and other diagnoses. Perceived healthcare services were assessed through questions on the use of treatments for endometriosis symptoms and sexual health, offering a multiple-choice list (eg, surgery, psychotherapy, physiotherapy) plus free-text options and expenditure estimates.

Adherence

Treatment adherence was defined as completing all eight modules. In the IG, dropout was defined as prolonged inactivity (>4 weeks) without response to reminders; since questionnaires were only released upon active use and completion of specific modules, dropouts did not receive any further assessments. In the CG, dropout was defined as non-response to the scheduled questionnaires despite reminder notifications. For each participant, the total time to complete all modules and the number of symptom-tracking entries during the intervention period were recorded as indicators of engagement. Within the *Odeya* symptom tracker, the IG could rate pain, stress, self-care, and sexual satisfaction on a 1–10 scale (higher scores = greater intensity), along with six standardized symptoms and any additional personally relevant symptoms.

Acceptance

Acceptance and user satisfaction were assessed with the *Client Satisfaction Questionnaire adapted to Internet-based* (CSQ-I), [13, 14] an eight-item measure of global satisfaction (score range 8–32). Items are rated on a 4-point Likert scale (1 = does not apply to me, 4 = totally applies to me), with higher total scores indicating greater satisfaction.

Usability was measured using the 18-item *German version of the mHealth App Usability Questionnaire* (G-MAUQ), covering three subscales: ease of use (5 items), interface and satisfaction (7 items), and usefulness (6 items). Items are rated on a 7-point Likert scale (1 = don't agree, 7 = agree), with higher mean scores indicating greater usability.[15]

Additionally, a Visual Analogue Scale (VAS; 1 = low satisfaction, 10 = high satisfaction) was used to assess overall satisfaction.[16]

Safety

Potential positive or negative effects were measured with the 25-item Inventory for the balanced assessment of Negative Effects of Psychotherapy – Online Intervention (INEP-ON),[17] using a bipolar 7-point scale measuring negative and positive effects (–3 to 3; Item 1-11) or a 4-point scale measuring only presence of negative effects (0 to 4; Item 12-23), plus two additional free text items assessing circumstances for life changes.

Additionally, participants were asked for any negative changes in health status or stressful life events during the study via free-text responses.

Sexual Health-Related Outcomes

Sexual distress

The *Female Sexual Distress Scale–Desire/Arousal/Orgasm* (FSDS-DAO)[18]15 items scored from 0 ("never") to 4 ("always"), with higher total scores indicating greater sexual distress (range 0–60). The questionnaire has been translated into German and validated by the research group (Kronthaler et al., manuscript submitted).

Sexual function

The *Screening for Sexual Problems* (SSP-F)[19] assesses based on ICD-11 criteria four domains of sexual function (desire, arousal, orgasm, pain) over the past 6 months, each scored from 0 ("never") to 4 ("always"); if a problem is reported (> 1), associated distress is rated from 0 ("not at all") to 4 ("very much"). Sexual dysfunction is present when distress in any domain is rated ≥ 3.

The *Female Sexual Function Index* (FSFI-d)[20] includes 19 items across six subscales (desire, arousal, lubrication, orgasm, satisfaction, pain; subscale range 0-6), each scored on a 5-point scale (except desire: 1–5), with higher scores indicating better sexual function (total range 2–36).

Sexual self-esteem

The *Sexual Self-Esteem Inventory*, short form (SSEI-d)[21] includes 35 items scored from 1 (“disagree strongly”) to 6 (“agree strongly”) on five subscales (skill/experience, attractiveness, control, moral judgement, adaptiveness; subscales range 1-6). Total average sum scores were calculated with higher mean scores indicating greater sexual self-esteem (range 1–6).

Fear and cognition around sexuality

The *Fear of Sexuality Questionnaire* (FSQ)[22] includes 8 items across two subscales (fear of non-coital sexual activity: range 5-25; fear of coitus: range 3-15), each scored from 1 (“never”) to 5 (“always”); higher total subscale scores indicate higher frequency of fear in sexual situations.

The *Vaginal Penetration Cognition Questionnaire* (VPCQ)[23] includes 22 items scored from 0 (“never”) to 6 (“always”) across five domains (control cognitions [reverse-scored], catastrophic and pain cognitions, self-image cognitions, positive cognitions, genital incompatibility cognitions). Higher mean scores on the catastrophic and pain cognitions, self-image cognitions, and genital incompatibility cognitions subscales indicate more negative cognitive responses, whereas higher mean scores on the positive cognitions and control cognitions (high levels of perceived penetration control) subscales indicate more positive cognitive responses.

The FSQ and the VPCQ were translated from Dutch/English into German by three independent native German speakers for use in the study.

Partnership

The *Partnership Questionnaire* (PFB)[24, 25] includes 30 items across three subscales (dispute behavior, tenderness, commonality/communication) plus one global satisfaction item; subscale items are scored from 0 (“never/very rarely”) to 3 (“very often”), and the global item from 0 (“very unhappy”) to 4 (“very happy”). High scores on dispute behavior indicate negative tendencies, while high scores on other subscales reflect positive behaviors. The total score is calculated as (30 − scale 1) + scale 2 + scale 3 (range 0–90), with scores ≤ 53 indicating an unsatisfied partnership. The questionnaire was administered only to women who reported being in a current partnership.

The *Questionnaire for the Assessment of Resources in Sexuality and Partnership* (RSP)[26] includes 25 items across five subscales (body perception, tenderness, desire, love, communication of sexual needs), each scored from 1 (“very often”) to 5 (“very rarely”). The total sum scores was calculated with lower scores indicating more positive relationship evaluations (range 25–125).

Overall Health-Related Outcomes

Overall health was assessed using the following validated self-report measures:

The *Childhood Trauma Questionnaire* (CTQ) [Klinitzke et al., 2012], includes 28 items across 5 subscales, rated from 1 (“never true”) to 5 (“very often true”). Subscale sum scores classify moderate to severe trauma as previous trauma experience.

The *Beck Depression Inventory II* (BDI-II)[27] consists of 21 items scored from 0 (no symptom) to 3 (severe symptom), with total score categories of 0–13 (minimal), 14–19 (mild), 20–28 (moderate), and 29–63 (severe depression).

The *Generalized Anxiety Disorder 7* (GAD-7)[28] contains 7 items scored from 0 (“not at all”) to 3 (“almost every day”), with total score categories of 0–4 (minimal), 5–9 (mild), 10–14 (moderate), and 15–21 (severe anxiety).

The *Brief Symptom Inventory* (BSI)[29, 30] comprises 53 items scored from 0 (“not at all”) to 4 (“extremely”), assessing nine symptom dimensions (Somatization, Obsession-Compulsion, Interpersonal Sensitivity, Depression, Anxiety, Hostility, Phobic anxiety, Paranoid ideation, Psychoticism) and three global distress indices (Global Severity Index, Positive Symptom

Distress Index, Positive Symptom Total) in last seven days, with raw scores converted to standardized T-scores.
The *Perceived Stress Scale 10* (PSS-10)[31] includes 10 items rated from 0 (“never”) to 4 (“very often”) with higher sum scores reflecting greater perceived stress in the past month (range 0-40).
The *Patient-Reported Outcomes Measurement Information System 29* (PROMIS-29)[32] includes 29 items covering seven domains (depression, anxiety, physical function, pain interference, fatigue, sleep disturbance, social participation), each scored from 1 to 5, with raw scores converted to standardized T-scores; an additional pain intensity scale is scored from 0 to 10.
The *European Health Interview Survey Quality of Life* (EUROHIS-QOL)[33] consists of 8 items scored from 0 (“not at all”) to 4 (“completely”), with higher sum scores indicating better quality of life (range 0-32).
The *Central Sensitization Inventory* (CSI-GE)[34] comprises 25 items scored from 0 (“never”) to 4 (“always”), with higher sum scores indicating greater symptom severity (range 1-100).
The *International Consultation on Incontinence Questionnaire Female Lower Urinary Tract Symptoms Modules* (ICIQ-FLUTS)[35] includes 12 items scored on a 5-point Likert scale ranging from 0 (no symptoms) to 4 (high symptom prevalence) and assesses symptoms on the three subscales: filling (range 0-16), voiding (range 0-12) and incontinence (range 0-20).

Qualitative Interviews

Sampling and Recruitment

IG Dropouts

All individuals who discontinued participation before completing the post-intervention assessment were invited to participate in this qualitative phase. The invitation process used multiple modes: participants could either respond to an online questionnaire in REDCap or choose to participate in a live interview via video call. The online questionnaire contained the same open-ended questions as the interview protocol to ensure comparability of responses. Only one participant opted for a live online interview; all others completed the questionnaire asynchronously. This flexible approach aligns with recommendations in dropout research to offer multiple response modalities in order to increase participation and reduce nonresponse bias.[36]

IG Completers

For the IG, only participants who had completed the full intervention program and all post-intervention assessments were eligible for the qualitative interviews. All eligible participants who expressed interest were invited to join focus groups. However, due to varying availability and the limited number of responses (n=3), we conducted individual interviews with these participants instead.

CG Completers

For the CG, participants were selected based on methodological recommendations to capture criterion sampling in demographic and contextual variables (age and relationship status).[37]

Questions

For the qualitative component, semi-structured guides were tailored to dropouts, intervention completers, and controls to capture subgroup-specific experiences and perspectives. Open-ended prompts allowed in-depth exploration while ensuring comparability. These guides were developed based on process evaluation frameworks for complex interventions,[38] see Appendix 3.

Qualitative Data Analysis

IG Dropouts

Free list interviews were analyzed in German using a collaborative online whiteboard platform (Miro),[39] which allows organizing and clustering qualitative data via digital sticky notes and has been applied in research contexts.[40] The free list method, a structured anthropological approach employing standardized questions to elicit list-based responses, enabled efficient qualitative data collection.[41, 42] Free listing is a qualitative elicitation method that rapidly yields structured, quantifiable data to understand how populations conceptualize and prioritize health-related domains. Participants are presented with open-ended prompts and asked to enumerate all items that come to mind. This approach harnesses spontaneous, unmediated responses grounded in participants' own language, making it particularly valuable for identifying community priorities, health behaviors, and perceived barriers. Lists are subsequently subjected to systematic data cleaning, whereby researchers iteratively combine synonyms, root words, and conceptually similar terms across participants.[43] Item responses are then quantified by frequency, that is, how many respondents mentioned each item. Responses from all interviews were compiled, categorized, and ranked by frequency to indicate thematic salience.[44] Data were transferred to Miro, with each response documented as a digital “post-it” labeled with a sequential participant ID distinct from the pseudonym to ensure analytic traceability. Categories were iteratively developed: one experienced researcher performed the initial coding, two co-investigators reviewed categorizations, and discrepancies were resolved through discussion until consensus was reached.

CG cand IG completers

Audio data were transcribed verbatim using *f4transkript*,[45] previously applied in German-language research.[46] A data-driven qualitative content analysis (QCA) based on Schreier’s toolbox model[47] was conducted using an abductive, primarily inductive approach. Initial coding by an experienced researcher was reviewed by a second researcher, with discrepancies resolved through discussion. Analysis was iterative, supported by MAXQDA (v24.3), and quality assurance ensured through audit trail, memos, and peer debriefing. Findings were translated into English with attention to preserving meaning and accuracy.

Results

Motivation and Expectations of Participation

Participants who took part in the qualitative interviews described a range of motivations for joining the study. Some expressed a sense of gratitude and emotional resonance with the topic:

> "I was really pleased that this topic is being researched, pleased in the sense of gratitude and feeling moved." (CG1)

As motivators, participants also described a need for self-care and curiosity about finding support:

> "I realized (…) I need to start taking care of myself." (IG1)
> "I'm definitely searching and curious to find some support." (CG1)

In terms of expectations, several participants reported having none, largely due to uncertainty about how a digital format might work:

> "None at all, because I just couldn't imagine how this would work via an app." (IG2)

Nonetheless, some expressed the hope to gain a better understanding of their condition:

> "I expected to develop more understanding [for my diagnose]." (IG3)

FSDS-DAO at Screening and Baseline in groups

Groups did not differ in FSDS-DAO scores at screening (CG: mean±SD, 34.90±9.09, IG: 37.76±10.62), or at baseline (T0, CG: 37.84±9.15, IG: 36.45±9.71). After being informed of their group assignment, the CG showed a notable mean worsening in sexual distress, reflected by a mean increase in FSDS-DAO scores of 2.94 points at T0 (95% CI [–5.63, –0.24]), whereas the IG showed no meaningful change (Δ=-1.31, 95% CI [–1.89, 4.52]).

Behavior Outside the App

Regarding healthcare behaviors, most IG participants reported no change in costs for endometriosis (90%, *n*=9/10) or sexuality (80%, *n*=8/10). Post-intervention, one IG participant began psychotherapy, and one CG participant began sex therapy, consistent with qualitative findings where one participant per group expressed interest in psychotherapy.

Willingness to Pay

Regarding the question if and how much they would pay after completing the intervention, the mean amount that women were willing to pay for the intervention was €83 (*n*=10, SD=€105.7, range €0–€300). Forty percent (*n*=4/10) preferred a one-time payment, while 30% (*n*=3/10) indicated they would not self-pay.

Safety

Balanced Effects: Negative Responses

Negative responses on balanced items were otherwise rare (≤10% at T2, <30% at T3). At T2, isolated concerns included more relationship conflicts (T2: *n*=1), worsened family relations (T2: *n*=1, T3: *n*=1), and insufficient moderator support (T2: *n*=1, T3: *n*=2). At T3, additional concerns included distress related to past experiences (*n*=2), worsened social relationships (*n*=1), loneliness (*n*=1), and reduced engagement in hobbies (*n*=1). Items addressing exclusively negative effects (see Table S10 in Appendix 2) showed longer

periods of not feeling well in 50% (n=5/10) at T2 and 71.4% (n=5/7) at T3. Further concerns included data protection (T2: n=3), partner problems (T2: n=2; T3: n=1), financial worries (T2: n=1; T3: n=1), negative self-perception (T2: n=1), and perceiving hurtful statements (T2: n=1; T3: n=1). Due to a coding error, attribution to lifestyle factors versus the intervention could not be determined.

References

1. Kosman E, Kronthaler S, Franck L, Mühle E, von der Reidt A, Vater M, et al. Addressing Sexual Distress in Gynecological Conditions: Development of a Digital Intervention. manuscript in preparation.
2. Richards DA, Bazeley P, Borglin G, Craig P, Emsley R, Frost J, et al. Integrating quantitative and qualitative data and findings when undertaking randomised controlled trials. BMJ Open. 2019 Nov 26;9(11):e032081. PMID: 31772096. doi: 10.1136/bmjopen-2019-032081.
3. Hwang Y, Knobf MT, Sadler LS. Integration in Mixed-Methods Research With an Exemplar Explanatory Sequential Study. Nurs Res. 2025 Mar–Apr 01;74(2):144–9. PMID: 39616427. doi: 10.1097/nnr.0000000000000796.
4. Zarski AC, Velten J, Knauer J, Berking M, Ebert DD. Internet- and mobile-based psychological interventions for sexual dysfunctions: a systematic review and meta-analysis. NPJ Digit Med. 2022 Sep 9;5(1):139. PMID: 36085306. doi: 10.1038/s41746-022-00670-1.
5. Frühauf S, Gerger H, Schmidt HM, Munder T, Barth J. Efficacy of psychological interventions for sexual dysfunction: a systematic review and meta-analysis. Arch Sex Behav. 2013 Aug;42(6):915–33. PMID: 23559141. doi: 10.1007/s10508-012-0062-0.
6. Pereira VM, Arias-Carrión O, Machado S, Nardi AE, Silva AC. Sex therapy for female sexual dysfunction. Int Arch Med. 2013 Sep 26;6(1):37. PMID: 24066697. doi: 10.1186/1755-7682-6-37.
7. Jaderek I, Lew-Starowicz M. A Systematic Review on Mindfulness Meditation-Based Interventions for Sexual Dysfunctions. J Sex Med. 2019 Oct;16(10):1581–96. PMID: 31570137. doi: 10.1016/j.jsxm.2019.07.019.
8. Basson R, Rees P, Wang R, Montejo AL, Incrocci L. Sexual function in chronic illness. J Sex Med. 2010 Jan;7(1 Pt 2):374–88. PMID: 20092445. doi: 10.1111/j.1743-6109.2009.01621.x.
9. Basson R. Sexual function of women with chronic illness and cancer. Womens Health (Lond). 2010 May;6(3):407–29. PMID: 20426607. doi: 10.2217/whe.10.23.
10. McCabe MP, Sharlip ID, Lewis R, Atalla E, Balon R, Fisher AD, et al. Risk Factors for Sexual Dysfunction Among Women and Men: A Consensus Statement From the Fourth International Consultation on Sexual Medicine 2015. J Sex Med. 2016 Feb;13(2):153–67. PMID: 26953830. doi: 10.1016/j.jsxm.2015.12.015.
11. McCool-Myers M, Theurich M, Zuelke A, Knuettel H, Apfelbacher C. Predictors of female sexual dysfunction: a systematic review and qualitative analysis through gender inequality paradigms. BMC Womens Health. 2018 Jun 22;18(1):108. PMID: 29929499. doi: 10.1186/s12905-018-0602-4.
12. Mitchell KR, Mercer CH, Ploubidis GB, Jones KG, Datta J, Field N, et al. Sexual function in Britain: findings from the third National Survey of Sexual Attitudes and Lifestyles (Natsal-3). Lancet. 2013 Nov 30;382(9907):1817–29. PMID: 24286787. doi: 10.1016/s0140-6736(13)62366-1.
13. Attkisson CC, Zwick R. The client satisfaction questionnaire. Psychometric properties and correlations with service utilization and psychotherapy outcome. Eval Program Plann. 1982;5(3):233–7. PMID: 10259963. doi: 10.1016/0149-7189(82)90074-x.
14. Boß L, Lehr D, Reis D, Vis C, Riper H, Berking M, et al. Reliability and Validity of Assessing User Satisfaction With Web-Based Health Interventions. J Med Internet Res. 2016 Aug 31;18(8):e234. PMID: 27582341. doi: 10.2196/jmir.5952.
15. Kopka M, Slagman A, Schorr C, Krampe H, Altendorf M, Balzer F, et al. German mHealth App Usability Questionnaire (G-MAUQ) and short version (G-MAUQ-S): Translation and validation study. Smart Health. 2024 2024/12/01/;34:100517. doi: https://doi.org/10.1016/j.smhl.2024.100517.
16. Hayes M, Patterson D. Experimental development of the graphic rating method. Psychological Bulletin. 1921;18:98–9.
17. Ladwig I, Rief W, Nestoriuc Y. Welche Risiken und Nebenwirkungen hat Psychotherapie? - Entwicklung des Inventars zur Erfassung Negativer Effekte von Psychotherapie (INEP). Verhaltenstherapie. 2014;24(4):252–63. doi: 10.1159/000367928.

18. Derogatis LR, Revicki DA, Rosen RC, Jordan R, Lucas J, Spana C. Psychometric validation of the Female Sexual Distress Scale-Desire/Arousal/Orgasm. J Patient Rep Outcomes. 2021 Sep 24;5(1):100. PMID: 34559353. doi: 10.1186/s41687-021-00359-1.
19. Velten J, Zarski A-C. Therapie-Tools Sexuelle Funktionsstörungen: Mit E-Book inside und Arbeitsmaterial. 1 ed. Weinheim: Beltz; 2022. ISBN: 978-3-621-28726-5.
20. Berner MM, Kriston L, Zahradnik HP, Härter M, Rohde A. Überprüfung der Gültigkeit und Zuverlässigkeit des deutschen Female Sexual Function Index (FSFI-d). Geburtshilfe Frauenheilkd. 2004 2004/03/11;64(03):293–303. doi: 10.1055/s-2004-815815.
21. Bornefeld-Ettmann P, Steil R, Höfling V, Weßlau C, Lieberz KA, Rausch S, et al. Validation of the German Version of the Sexual Self-Esteem Inventory for Women and its Application in a Sample of Sexually and Physically Abused Women. Sex Roles. 2018 2018/07/01;79(1):109–22. doi: 10.1007/s11199-017-0849-5.
22. Ter Kuile MM, Melles R, de Groot HE, Tuijnman-Raasveld CC, van Lankveld J. Therapist-aided exposure for women with lifelong vaginismus: a randomized waiting-list control trial of efficacy. J Consult Clin Psychol. 2013 Dec;81(6):1127–36. PMID: 24060195. doi: 10.1037/a0034292.
23. Klaassen M, Ter Kuile MM. Development and initial validation of the vaginal penetration cognition questionnaire (VPCQ) in a sample of women with vaginismus and dyspareunia. J Sex Med. 2009 Jun;6(6):1617–27. PMID: 19538428. doi: 10.1111/j.1743-6109.2009.01217.x.
24. Hahlweg K. Fragebogen zur Partnerschaftsdiagnostik (PFB) [Partnership Questionnaire]. Göttingen: Hogrefe; 1996.
25. Hinz A, Stöbel-Richter Y, Brähler E. Der Partnerschaftsfragebogen (PFB). Diagnostica. 2001 2001/07/01;47(3):132–41. doi: 10.1026//0012-1924.47.3.132.
26. Klingler OJ, Loewit KK. Der Fragebogen „Ressourcen in Sexualität und Partnerschaft“ (RSP) – Konzeption und erste Ergebnisse zur Validität. Zeitschrift für Differentielle und Diagnostische Psychologie. 1996;17(4):268–75.
27. Beck ATS, R. A.; Brown, G. K. Beck Depression Inventory (BDI-II). 2nd ed. San Antonio, TX: Pearson; 1996. ISBN: 9780158018089.
28. Spitzer RL, Kroenke K, Williams JB, Löwe B. A brief measure for assessing generalized anxiety disorder: the GAD-7. Arch Intern Med. 2006 May 22;166(10):1092–7. PMID: 16717171. doi: 10.1001/archinte.166.10.1092.
29. Derogatis LR. Brief Symptom Inventory (BSI): Administration, scoring, and procedures manual. 3rd ed. Minneapolis, MN: National Computer Systems; 1993.
30. Geisheim C, Hahlweg K, Fiegenbaum W, Frank M, Schröder B, von Witzleben I. Das Brief Symptom Inventory (BSI) als Instrument zur Qualitätssicherung in der Psychotherapie. Diagnostica. 2002 2002/01/01;48(1):28–36. doi: 10.1026//0012-1924.48.1.28.
31. Klein EM, Brähler E, Dreier M, Reinecke L, Müller KW, Schmutzer G, et al. The German version of the Perceived Stress Scale – psychometric characteristics in a representative German community sample. BMC Psychiatry. 2016 2016/05/23;16(1):159. doi: 10.1186/s12888-016-0875-9.
32. Cella D, Yount S, Rothrock N, Gershon R, Cook K, Reeve B, et al. The Patient-Reported Outcomes Measurement Information System (PROMIS): progress of an NIH Roadmap cooperative group during its first two years. Med Care. 2007 May;45(5 Suppl 1):S3–S11. PMID: 17443116. doi: 10.1097/01.mlr.0000258615.42478.55.
33. Schmidt S, Mühlan H, Power M. The EUROHIS-QOL 8-item index: psychometric results of a cross-cultural field study. Eur J Public Health. 2006 Aug;16(4):420–8. PMID: 16141303. doi: 10.1093/eurpub/cki155.
34. Klute M, Laekeman M, Kuss K, Petzke F, Dieterich A, Leha A, et al. Cross-cultural adaptation and validation of the German Central Sensitization Inventory (CSI-GE). BMC Musculoskelet Disord. 2021 Aug 18;22(1):708. PMID: 34407773. doi: 10.1186/s12891-021-04481-5.
35. Brookes ST, Donovan JL, Wright M, Jackson S, Abrams P. A scored form of the Bristol Female Lower Urinary Tract Symptoms questionnaire: data from a randomized controlled trial of surgery for women with stress incontinence. Am J Obstet Gynecol. 2004 Jul;191(1):73–82. PMID: 15295345. doi: 10.1016/j.ajog.2003.12.027.
36. Rybak A. Survey mode and nonresponse bias: A meta-analysis based on the data from the international social survey programme waves 1996–2018 and the European social survey rounds 1 to 9. PLOS ONE. 2023;18(3):e0283092. doi: 10.1371/journal.pone.0283092.

37. Patton MQ. Qualitative Research & Evaluation Methods: Integrating Theory and Practice. 4th ed. ed. Thousand Oaks, CA: SAGE Publications; 2015. ISBN: 9781483301457.
38. Moore GF, Audrey S, Barker M, Bond L, Bonell C, Hardeman W, et al. Process evaluation of complex interventions: Medical Research Council guidance. Bmj. 2015 Mar 19;350:h1258. PMID: 25791983. doi: 10.1136/bmj.h1258.
39. Miro. Miro. Amsterdam, Netherlands: Miro; 2025.
40. Blair C, Best P, Burns P, Campbell A, Davidson G, Duffy J, et al. 'Getting involved in research': a co-created, co-delivered and co-analysed course for those with lived experience of health and social care services. Res Involv Engagem. 2022 May 16;8(1):20. PMID: 35578275. doi: 10.1186/s40900-022-00353-x.
41. Burchert S, Alkneme MS, Bird M, Carswell K, Cuijpers P, Hansen P, et al. User-Centered App Adaptation of a Low-Intensity E-Mental Health Intervention for Syrian Refugees. Front Psychiatry. 2018;9:663. PMID: 30740065. doi: 10.3389/fpsyt.2018.00663.
42. Decker-Palmer M, Klodowski D, Thompson T, Lanoue M, Messina A, Schroeder D, et al. Freelisting: A Technique for Enhancing the Community Health Needs Assessment. Community Health Equity Res Policy. 2024 Jan;44(2):201–8. PMID: 36513364. doi: 10.1177/2752535x221146232.
43. Keddem S, Barg FK, Frasso R. Practical Guidance for Studies Using Freelisting Interviews. Prev Chronic Dis. 2021 Jan 14;18:E04. PMID: 33444525. doi: 10.5888/pcd17.200355.
44. Applied Mental Health Research Group. Design, Implementation, Monitoring, and Evaluation of Mental Health and Psychosocial Assistance Programs for Trauma Survivors in Low Resource Countries: A User's Manual for Researchers and Program Implementers. Module 1: Qualitative Assessment (Adult Version). Baltimore, MD: 2013.
45. f4transkript. f4transkript. Marburg, Germany: audiotranskription; 2025.
46. Wollin-Giering S, Hoffmann M, Höfting J, Ventzke C. Automatic Transcription of English and German Qualitative Interviews. Forum Qualitative Sozialforschung / Forum: Qualitative Social Research. 2024 01/29;25(1). doi: 10.17169/fqs-25.1.4129.
47. Schreier M. Ways of Doing Qualitative Content Analysis: Disentangling Terms and Terminologies. Forum Qualitative Sozialforschung / Forum: Qualitative Social Research. 2014 01/19;15(1). doi: 10.17169/fqs-15.1.2043.

Table S1. Schedule of assessments and quantitative outcome measures by assessment timepoint.

Variables	Screening	T0	T1	T2	T3
**Demographics**					
Sociodemographic measurement	x	-	-	-	-
Medical history	x	-	x	x	x
Perceived services in the healthcare system	x	-	x	x	x
**Adherence**					
Dropout, engagement	-	x	x	x	x
**Acceptance**					
Client Satisfaction (VAS)	-	-	x	x	-
Client Satisfaction Questionnaire – Internet (CSQ-I)	-	-	-	x	-
German version of the mHealth App Usability Questionnaire (G-MAUQ)	-	-	-	x	-
**Safety**					
Inventory for the Balanced Assessment of Negative Effects of Psychotherapy – Online Intervention (INEP-ON)	-	-	-	x	x
Changes in health status	-	x	x	x	-
**Sexual Health-Related Outcomes**					
Female Sexual Distress Scale (FSDS-DAO)	x	x	x	x	x
Screening for Sexual Problems (SSP-F)	x				
Female Sexual Function Index (FSFI-d)	-	x	x	x	x
Sexual Self-Esteem-Inventory (SSEI-d)	-	x	x	x	x
Fear of Sexuality Questionnaire (FSQ)	-	x	x	x	x
Vaginal Penetration Cognition Questionnaire (VPCQ)	-	x	x	x	x
Partnership questionnaire (PFB)	-	x	x	x	x
Questionnaire of resources in sexuality and partnership (RSP)	-	x	x	x	x
Central Sensitization Inventory (CSI-GE)	-	x	-	x	x
**Overall Health-Related Outcomes**					
Childhood Trauma Questionnaire (CTQ)	x	-	-	-	-
Beck-Depressions-Inventor (BDI-II)	x	-	-	x	-
Generalized Anxiety Disorder (GAD-7)	x	-	-	x	-
Brief Symptom Inventory (BSI)	x	-	-	x	-
Perceived Stress Scale (PSS-10)	-	x	x	x	x
Patient-Reported Outcome Measurement Information System (PROMIS-29)	-	x	x	x	x
European Health Interview Survey-Quality of Life (EUROHIS-QOL)	-	x	-	x	x
International Consultation on Incontinence Questionnaire Female Lower Urinary Tract Symptoms Modules (ICIQ-FLUTS)	-	x	x	x	x

T0, baseline. T1, after Module 5/5 weeks after baseline. T2, after Module 8/8 weeks after baseline. T3, 6-month follow-up after baseline.

Table S2. Characteristics of Qualitative Assessment Participants (*N*=16) at Baseline (T0).

Characteristic	IG Completer (*n*=3)	CG Completer (*n*=2)	IG Dropout (*n*=11)
Age (y), M (SD)	33.67 (5.69)	37.50 (13.44)	29.36 (3.91)
In relationship, n (%)	3 (100)	1 (50)	10 (90.9)
Relationship duration in months, M (SD)	134.33 (135.89)	90 (NA)	65.33 (50.65)
Relationship satisfaction (0–10), Md (IQR)	6.0 (5.0–7.0)	10.0 (10.0–10.0)	8.0 (8.0-9.0)
Education 12 years, *n* (%)	1 (33.3)	2 (100.0)	8 (72.7)
Urban Residence, *n* (%)	1 (33.3)	2 (100.0)	7 (63.6)
Heterosexual, *n* (%)	3 (100.0)	1 (50.0)	8 (72.7)
Partnered intimacy [a], *n* (%)	2 (66.7)	1 (50.0)	10 (90.9)
Masturbation [a], *n* (%)	0 (0.0)	0 (0.0)	4 (36.4)
Religious, *n* (%)	1 (33.3)	0 (0.0)	5 (45.5)
Histologically confirmed Endometriosis, *n* (%)	2 (66.7)	0 (0.0)	10 (90.9)
Operation, *n* (%)	2 (66.7)	0 (0.0)	11 (100)
Hormonal medication, *n* (%)			
Progestin-only contraceptive pill	1 (33.3)	1 (50)	5 (45.5)
Combined oral contraceptive pill	0 (0.0)	0 (0.0)	2 (18.2)
Sex therapy or Couples therapy, *n* (%)	0 (0.0)	1 (50)	0 (0.0)
Psychotherapy, *n* (%)	2 (66.7)	2 (100)	7 (63.6)
Other diagnoses, *n* (%)			
PCOS	0 (0.0)	0 (0.0)	0 (0.0)
PMS	1 (33.3)	1 (50)	2 (18.2)
Uterus myomatosus	0 (0.0)	0 (0.0)	1 (9.1)
Uterus prolapse	0 (0.0)	0 (0.0)	0 (0.0)
Incontinence	0 (0.0)	0 (0.0)	1 (9.1)
Infertility	0 (0.0)	0 (0.0)	0 (0.0)
Vulvodynia	0 (0.0)	0 (0.0)	0 (0.0)
Lichen sclerosus	0 (0.0)	0 (0.0)	0 (0.0)
Cancer	0 (0.0)	0 (0.0)	0 (0.0)
Lifestyle, n (%)			
Physical activity	2 (66.7)	2 (100.0)	8 (72.7)
Healthy diet	3 (100.0)	2 (100.0)	10 (90.9)
Smoking	0 (0.0)	1 (50.0)	1 (9.1)
Alcohol consumption	0 (0.0)	1 (50.0)	2 (18.2)
BDI-II (0–63), M (SD)	17.33 (6.35)	16 (2.83)	15.91 (6.09)
GAD-7 (0–21), M (SD)	7.67 (6.03)	7 (0.0)	7.91 (3.48)
SSP-F, n (%)	3 (100.0)	2 (100.0)	8 (72.73)
BSI GSI, M (SD)	61.67 (14.29)	55 (18.38)	59 (11.62)
BSI PSDI, M (SD)	56.33 (16.5)	54 (5.66)	52.27 (11.33)
BSI PST, M (SD)	65 (15.1)	60.5 (26.16)	62 (11.98)
CTQ: Sexual abuse, *n* (%)	0 (0.0)	1 (50.0)	3 (27.3)
CTQ: Any trauma [c], *n* (%)	0 (0.0)	1 (50.0)	5 (45.5)

Abbreviation: IG, intervention group. Partnered intimacy referred to activities such as cuddling and kissing. PCOS, polycystic ovary syndrome. PMS, premenstrual syndrome. BDI-II, Beck-Depression-Inventory-II. GAD-7, Generalized Anxiety Disorder Scale-7. SSP-F, Screening for Sexual Problems. BSI, Brief Symptom Inventory. GSI, Global Severity Index. PSDI, Positive Symptom Distress Index. PST, Positive Symptom Total. CTQ, Childhood Trauma Questionnaire.

[a] >once per week.

[b] Sexual trauma was defined using the CTQ Sexual Abuse Subscale, with a cutoff score of 8 [Klinitzke et al., 2012].

[c] Any trauma was defined as meeting the CTQ cutoff for at least moderate severity [Klinitzke et al., 2012].

Table S3. Characteristics of Intervention Completers and Dropouts at Baseline (T0).

**Characteristic**	**Completer (*n*=10)**	**Dropout (*n*=19)**
Age (y), M (SD)	33 (7.18)	30.11 (3.84)
In relationship, n (%)	9 (90.0)	17 (89.5)
Relationship duration in months, M (SD)	87.22 (78.98)	59.56 (39.39)
Relationship satisfaction (0–10), Md (IQR)	8.0 (7.0-9.0)	8.0 (8.0-9.0)
Education 12 years, *n* (%)	8 (80.0)	15 (78.9)
Urban Residence, *n* (%)	6 (60.0)	10 (52.6)
Heterosexual, *n* (%)	8 (80.0)	15 (78.9)
Partnered intimacy [a], *n* (%)	8 (80.0)	16 (84.2)
Masturbation [a], *n* (%)	1 (10.0)	6 (31.6)
Religious, *n* (%)	2 (20.0)	8 (42.1)
Histologically confirmed Endometriosis, *n* (%)	8 (80.0)	18 (94.7)
Operation, *n* (%)	7 (70.0)	19 (100.0)
Hormonal medication, *n* (%)		
Progestin-only contraceptive pill	3 (30.0)	7 (36.8)
Combined oral contraceptive pill	0 (0.0)	2 (10.5)
Sex therapy or Couples therapy, *n* (%)	0 (0.0)	0 (0.0)
Psychotherapy, *n* (%)	8 (80.0)	9 (47.4)
Other diagnoses, *n* (%)		
PCOS	0 (0.0)	0 (0.0)
PMS	2 (20.0)	6 (31.6)
Uterus myomatosus	0 (0.0)	3 (15.8)
Uterus prolapse	0 (0.0)	0 (0.0)
Incontinence	0 (0.0)	1 (5.3)
Infertility	0 (0.0)	1 (5.3)
Vulvodynia	0 (0.0)	0 (0.0)
Lichen sclerosus	0 (0.0)	0 (0.0)
Cancer	1 (10.0)	1 (5.3)
Lifestyle, n (%)		
Physical activity	9 (90.0)	13 (68.4)
Healthy diet	9 (90.0)	16 (84.2)
Smoking	0 (0.0)	2 (10.5)
Alcohol consumption	0 (0.0)	3 (15.8)
BDI-II (0–63), M (SD)	10.7 (7.7)	14.89 (6.31)
GAD-7 (0–21), M (SD)	5.5 (3.84)	7.11 (3.63)
SSP-F, n (%)	10 (100.00)	15 (78.95)
BSI GSI, M (SD)	53.1 (12.91)	55.79 (12.9)
BSI PSDI, M (SD)	52 (12.88)	55.84 (10.56)
BSI PST, M (SD)	53.6 (12.76)	56.42 (13.31)
CTQ: Sexual abuse, *n* (%)	0 (0.0)	3 (15.8)
CTQ: Any trauma [c], *n* (%)	1 (10.0)	8 (42.1)

Abbreviation: IG, intervention group. Partnered intimacy referred to activities such as cuddling and kissing. PCOS, polycystic ovary syndrome. PMS, premenstrual syndrome. BDI-II, Beck-Depression-Inventory-II. GAD-7, Generalized Anxiety Disorder Scale-7. SSP-F, Screening for Sexual Problems. BSI, Brief Symptom Inventory. GSI, Global Severity Index. PSDI, Positive Symptom Distress Index. PST, Positive Symptom Total. CTQ, Childhood Trauma Questionnaire.

[a] >once per week.

[b] Sexual trauma was defined using the CTQ Sexual Abuse Subscale, with a cutoff score of 8 [Klinitzke et al., 2012].

[c] Any trauma was defined as meeting the CTQ cutoff for at least moderate severity [Klinitzke et al., 2012].

Table S4. Screening and Baseline Values of Outcomes Stratified for IG Completers and Dropouts.

		Screening: M (SD)		T0: M (SD)	
**Questionnaires**		**Completer (n=10)**	**Dropout (n=19)**	**Completer (n=10)**	**Dropout (n=19)**
FSDS-DAO	Mean (SD)	37.20 (8.48)	38.05 (11.79)	37.90 (7.67)	35.68 (10.74)
	Md (IQR)	37.50 (13.50)	37.00 (16.50)	40.00 (7.50)	34.00 (11.50)
FSFI-d					
Total	Mean (SD)	-	-	14.35 (6.54)	18.07 (7.63)
	Md (IQR)	-	-	15.20 (7.75)	21.00 (12.55)
Desire	Mean (SD)	-	-	1.92 (0.79)	2.46 (1.00)
	Md (IQR)	-	-	1.80 (1.20)	2.40 (1.50)
Arousal	Mean (SD)	-	-	2.49 (1.41)	2.91 (1.66)
	Md (IQR)	-	-	2.55 (2.10)	3.60 (2.40)
Lubrication	Mean (SD)	-	-	3.54 (1.88)	3.76 (1.87)
	Md (IQR)	-	-	3.75 (2.77)	3.90 (2.10)
Orgasm	Mean (SD)	-	-	2.48 (1.73)	3.85 (2.18)
	Md (IQR)	-	-	2.40 (2.00)	4.00 (3.80)
Pain	Mean (SD)	-	-	1.32 (1.27)	1.77 (1.36)
	Md (IQR)	-	-	1.40 (2.30)	1.60 (2.00)
Satisfaction	Mean (SD)	-	-	2.60 (1.30)	3.33 (1.30)
	Md (IQR)	-	-	2.80 (1.90)	3.20 (1.80)
SSEI-d					
Total	Mean (SD)	-	-	4.00 (0.68)	3.96 (0.62)
	Md (IQR)	-	-	3.93 (0.63)	3.99 (0.90)
Skill & Experience	Mean (SD)	-	-	3.21 (0.87)	3.40 (0.80)
	Md (IQR)	-	-	3.29 (1.43)	3.43 (1.29)
Attractiveness	Mean (SD)	-	-	4.41 (1.10)	4.13 (0.98)
	Md (IQR)	-	-	4.71 (1.50)	4.21 (1.07)
Control	Mean (SD)	-	-	4.17 (0.76)	4.13 (0.98)
	Md (IQR)	-	-	4.14 (1.07)	4.36 (1.39)
Moral Judgement	Mean (SD)	-	-	4.77 (0.70)	4.53 (0.61)
	Md (IQR)	-	-	4.86 (0.79)	4.64 (0.93)
Adaptiveness	Mean (SD)	-	-	3.43 (1.15)	3.62 (0.98)
	Md (IQR)	-	-	3.36 (1.07)	3.36 (1.14)
FSQ					
Fear of Non-Coital Activity	Mean (SD)	-	-	13.30 (3.97)	11.67 (3.56)
	Md (IQR)	-	-	12.00 (5.75)	11.00 (4.50)
Fear of Coitus	Mean (SD)	-	-	9.70 (3.40)	8.78 (2.53)
	Md (IQR)	-	-	9.00 (3.25)	9.00 (3.00)
VPCQ					
Control Cognitions	Mean (SD)	-	-	4.30 (1.21)	4.24 (1.37)
	Md (IQR)	-	-	4.88 (1.88)	4.12 (2.44)
Catastrophic and Pain Cognitions	Mean (SD)	-	-	3.28 (1.32)	3.08 (1.23)
	Md (IQR)	-	-	3.30 (1.80)	3.10 (1.55)
Self-Image Cognitions	Mean (SD)	-	-	1.83 (0.60)	2.10 (0.87)
	Md (IQR)	-	-	1.83 (0.88)	2.08 (0.63)
Positive Cognitions	Mean (SD)	-	-	2.90 (1.10)	3.10 (1.32)
	Md (IQR)	-	-	3.20 (1.30)	3.40 (1.70)
Incompatibility	Mean (SD)	-	-	1.90 (1.98)	1.47 (1.27)
	Md (IQR)	-	-	1.75 (3.00)	1.75 (2.25)
PFB					
Total	mean (SD)	-	-	72.00 (15.17)	71.59 (11.77)
	Md (IQR)	-	-	77.00 (11.00)	71.00 (14.00)
Dispute Behavior	mean (SD)	-	-	2.44 (3.54)	4.29 (4.04)
	Md (IQR)	-	-	2.00 (3.00)	3.00 (6.00)
Tenderness	mean (SD)	-	-	21.56 (7.92)	22.82 (4.81)
	Md (IQR)	-	-	24.00 (8.00)	22.00 (6.00)
Communication	mean (SD)	-	-	22.89 (6.23)	23.06 (5.20)
	Md (IQR)	-	-	23.00 (11.00)	24.00 (6.00)
RSP					
Total	Mean (SD)	-	-	66.70 (18.96)	58.39 (14.83)
	Md (IQR)	-	-	65.00 (23.50)	59.50 (12.50)
Body Perception	Mean (SD)	-	-	13.70 (5.56)	12.78 (3.87)
	Md (IQR)	-	-	14.00 (8.75)	13.00 (2.75)
Tenderness	Mean (SD)	-	-	9.90 (5.40)	8.50 (2.85)
	Md (IQR)	-	-	8.50 (5.50)	8.50 (3.75)
Desire	Mean (SD)	-	-	19.70 (5.23)	16.83 (5.11)
	Md (IQR)	-	-	20.50 (6.75)	18.00 (7.50)

Love	Mean (SD)	-	-	8.80 (2.49)	8.78 (2.94)
	Md (IQR)	-	-	9.00 (2.50)	8.50 (3.75)
Communication	Mean (SD)	-	-	14.60 (4.99)	11.50 (4.20)
	Md (IQR)	-	-	14.50 (2.50)	11.50 (3.75)
CSI-GE	Mean (SD)	-	-	41.40 (15.94)	45.16 (15.12)
	Md (IQR)	-	-	45.00 (20.25)	47.00 (12.00)
BDI-II	Mean (SD)	10.70 (7.70)	14.89 (6.31)	-	-
	Md (IQR)	12.00 (10.00)	15.00 (10.00)	-	-
GAD-7	Mean (SD)	5.50 (3.84)	7.11 (3.63)	-	-
	Md (IQR)	4.50 (5.50)	7.00 (3.50)	-	-
PPS-10	Mean (SD)	-	-	18.80 (5.16)	21.00 (4.85)
	Md (IQR)	-	-	18.50 (5.50)	20.50 (5.50)
PROMIS-29					
Depression	Mean (SD)	-	-	57.52 (10.37)	63.09 (6.93)
	Md (IQR)	-	-	55.30 (14.68)	62.50 (5.20)
Anxiety	Mean (SD)	-	-	56.45 (9.74)	60.02 (6.29)
	Md (IQR)	-	-	56.45 (8.05)	59.50 (10.27)
Physical Function	Mean (SD)	-	-	59.17 (5.28)	60.57 (7.85)
	Md (IQR)	-	-	58.50 (7.17)	61.85 (11.00)
Pain Interference	Mean (SD)	-	-	59.17 (5.28)	60.57 (7.85)
	Md (IQR)	-	-	58.50 (7.17)	61.85 (11.00)
Pain Intensity	Mean (SD)	-	-	4.30 (1.95)	5.50 (1.79)
	Md (IQR)	-	-	5.00 (1.00)	6.00 (2.75)
Fatigue	Mean (SD)	-	-	60.20 (7.94)	66.19 (7.42)
	Md (IQR)	-	-	57.00 (11.20)	68.80 (12.57)
Sleep disturbance	Mean (SD)	-	-	53.62 (6.59)	58.79 (7.43)
	Md (IQR)	-	-	52.30 (5.60)	58.95 (13.18)
Social participation	Mean (SD)	-	-	46.08 (5.74)	45.99 (6.33)
	Md (IQR)	-	-	46.80 (7.43)	43.90 (8.33)
EUROHIS-QOL	Mean (SD)	-	-	28.70 (4.16)	23.95 (7.13)
	Md (IQR)	-	-	27.50 (6.25)	24.00 (6.00)
ISIQ-FLUTS					
Total	Mean (SD)	-	-	6.50 (4.03)	9.28 (4.23)
	Md (IQR)	-	-	7.00 (2.50)	9.50 (5.75)
Filling symptoms	Mean (SD)	-	-	3.30 (2.16)	4.67 (2.11)
	Md (IQR)	-	-	3.50 (2.00)	5.50 (3.50)
Voiding symptoms	Mean (SD)	-	-	2.40 (3.20)	2.72 (1.84)
	Md (IQR)	-	-	1.50 (3.50)	2.00 (2.75)
Incontinence symptoms	Mean (SD)	-	-	0.80 (1.55)	1.89 (3.12)
	Md (IQR)	-	-	0.00 (1.00)	0.00 (2.75)

Abbreviation: IG, intervention group. T0, baseline. FSDS-DAO, Female Sexual Distress Scale-Desire/Arousal/Orgasm. FSFI, Female Sexual Function Index. SSEI-d, Sexual Self-Esteem-Inventory (German version). FSQ, Fear of Sexuality Questionnaire. VPCQ, Vaginal Penetration Cognition Questionnaire. PFB, Partnership Questionnaire. RSP, Resources in Sexuality and Partnership. CSI-GE, Central Sensitization Inventory (German version). BDI-II, Beck-Depression-Inventory-II. GAD-7, Generalized Anxiety Disorder Scale-7. PPS-10, Perceived Stress Scale. PROMIS-29, Patient-Reported Outcomes Measurement Information System-29. EUROHIS-QOL, European Health Interview Survey-Quality of Life. ISIQ-FLUTS, International Consultation on Incontinence Questionnaire Female Lower Urinary Tract Symptoms Modules.

Table S5. Dropout Interview Question 1 (n=11).

**Factors leading to the discontinuation of app usage**		
Problem	Description of the category	Frequency n (%)
Time constraints	Daily responsibilities, stress, and life events (e.g., work, school, illness) limit capacity to continue the app usage.	6 (55)
Technical difficulties	App malfunctions and access issues (e.g., blocked installation, input limitations) disrupted participants' usage behavior.	3 (27)
Life changes	Changes in private life, such as relocation, job changes or personal crises (e.g., job loss due to illness) disrupted routines and prevented continuation of app usage.	2 (18)
Pain	Pain episodes limited the ability to manage daily life and left no capacity for additional tasks such as app usage.	2 (18)
Depression	Depression led to loss of motivation and energy, preventing continued app usage.	2 (18)
Symptom improvement outside of app usage	A significant symptom relief and regained sexual well-being was experienced (e.g., after surgery and physical healing), reducing the perceived need for further support through the app.	2 (18)
App content too time-consuming for daily life	Exercises were perceived as too long to continue app usage.	2 (18)
**Others:**		7 (64)
Relationship breakup		1 (9)
Difficulty with concentration		1 (9)
Problems with vaginal flora		1 (9)
Miscommunication with investigator		1 (9)
Insufficient time alone for app usage		1 (9)
Actors' credibility		1 (9)
App as an unsuitable format		1 (9)

Table S6. Dropout Interview Question 2 (n=11).

**Measures to prevent discontinuation of app usage**		
Problem	Description of the category	Frequency *n* (%)
Length of app units	Shorter units would be preferred.	2 (18)
No changes to the app necessary	It was emphasized that the discontinuation of app use was due to personal circumstances rather than app-related issues.	2 (18)
**Others:**		6 (55)
Getting more app usage time		1 (9)
Freely selectable topics and optional text fields		1 (9)
Solving technical problems		1 (9)
Restructuring of content		1 (9)
Clear communication on the part of the investigators		1 (9)
More credible content		1 (9)

Table S7. Dropout Interview Question 3 (n=11).

**Factors that could have motivated continued usage of the app**		
Problem	Description of the category	Frequency *n* (%)
No additional factors needed	The app was positively received.	2 (18)
**Others:**		5 (72)
Read aloud function of the texts		1 (9)
Reminders and the possibility to collect streaks		1 (9)
More flexibility in app usage time		1 (9)
Shorter units		1 (9)
Possibility to fill out the symptom tracker retrospectively		1 (9)

Table S8. Descriptive Statistics for Acceptance of the intervention at T2 (*n*=10).

Questionnaire	M	SD	Md	Min	Max
CSQ-I (range 8-32)	26.60	4.12	27.00	19.00	32.00
Items 1, 2, 3, 5, 7*: degree to which the intervention fulfilled their general satisfaction with the quality, kind of training, and amount of help they received*	16.30	2.45	16.00	13.00	20.00
Item 4*: degree to which respondents would recommend the intervention to others*	3.50	0.71	4.00	2.00	4.00
Item 6*: degree to which the intervention helped them to deal with their problems*	3.10	0.88	3.00	2.00	4.00
Item 8*: likelihood of using the intervention for themselves again*	3.70	0.67	4.00	2.00	4.00
G-MAUQ (range 1-7)	5.38	0.74	5.61	4.33	6.28
Ease of use	6.46	0.64	6.70	5.00	7.00
Interface satisfaction	5.28	0.78	5.12	4.38	7.00
Usefulness	4.46	1.69	4.70	1.60	6.80
VAS for satisfaction (higher values indicate higher satisfaction, range 0-10)	7.00	1.83	7.50	3.00	9.00

Abbreviation. CSQ-I, Client Satisfaction Questionnaire–Internet. G-MAUQ, German mHealth App Usability Questionnaire. VAS, Visual Analog Scale, M, Mean. SD, Standard Deviation. Md, Median. Min, Minimum. Max, Maximum. T2, after Module 8 of the intervention.

Table S9. INEP-ON Balanced Changes.

	**T2 (*n*=10)**			**T3 (*n*=7)**		
**Item**	Positive *n* (%)	Negative *n* (%)	No change, *n* (%)	Positive *n* (%)	Negative *n* (%)	No change, *n* (%)
INEP1	9 (90)	0 (0.0)	1 (10)	2 (28.6)	4 (57.1)	1 (14.3)
INEP2	4 (40	0 (0.0)	6 (60)	2 (28.6)	0 (0.0)	5 (71.4)
INEP 3	6 (60)	0 (0.0)	4 (40)	1 (14.3)	2 (28.6)	4 (57.1)
INEP 4	4 (40)	1 (10)	4 (40)	4 (57.1)	0 (0.0)	2 (28.6)
INEP 5	1 (50)	1 (10)	8 (80)	1 (14.3)	1 (14.3)	5 (71.4)
INEP 6	2 (20)	0 (0.0)	8 (80)	1 (14.3)	1 (14.3)	5 (71.4)
INEP 7	2 (20)	0 (0.0)	8 (80)	1 (14.3)	2 (28.6)	4 (57.1)
INEP 8	1 (10)	0 (0.0)	9 (90)	0 (0.0)	2 (28.6)	5 (71.4)
INEP 9	10 (100)	0 (0.0)	0 (0.0)	6 (85.7)	0 (0.0)	1 (14.3)
INEP 10	6 (60)	0 (0.0)	4 (40)	3 (42.9)	0 (0.0)	4 (57.1)
INEP 11	9 (90)	1 (10)	0 (0.0)	4 (57.1)	2 (28.6)	1 (14.3)

Abbreviation. INEP-ON, Inventory for the Balanced Assessment of Negative Effects of Psychotherapy – Online Intervention. T2, after Module 8. T3, 6-month follow-up after baseline.

Table S10. INEP-ON Negative Changes.

	**T2 (*n*=10)**		**T3 (*n*=7)**	
**Item**	≥1 (*n*)	≥1 (%)	≥1 (*n*)	≥1 (%)
INEP12	0	0.0	0	0.0
INEP13	0	0.0	0	0.0
INEP14	1	10	1	14.3
INEP15	0	0.0	0	0.0
INEP16	0	0.0	0	0.0
INEP17	2	20	1	14.3
INEP18	5	50	5	71.4
INEP19	1	10	0	0.0
INEP20	0	0.0	0	0.0
INEP21	0	0.0	0	0.0
INEP22	1	10	1	14.3
INEP23	3	30	0	0.0

Abbreviation. INEP-ON, Inventory for the Balanced Assessment of Negative Effects of Psychotherapy – Online Intervention. T2, after Module 8. T3, 6-month follow-up after baseline.

Table S11. Mean Scores and Standard Deviation at Baseline (T0), Mid-Intervention (T1), Post-Intervention (T2), and 3-Month-Post Intervention (T3), Stratified by Group.

		**T0**		**T1**		**T2**		**T3**	
**Questionnaires**		**IG**	**CG**	**IG**	**CG**	**IG**	**CG**	**IG**	**CG**
**Sexual Health-Related Outcomes**									
FSDS-DAO	*n*	29	31	15	28	10	24	8	24
	Mean (SD)	36.45 (9.71)	37.84 (9.15)	26.93 (12.56)	33.79 (13.8)	25.2 (16.21)	22.25 (13.79)	19.75 (17.77)	30.33 (13.79)
	Md (IQR)	36 (31–42)	36 (32.5–45)	29 (18.5–34.5)	35 (27.5–44)	17.5 (13.5–34)	24.5 (11–33.75)	16.5 (10–21.75)	29.5 (24–42)
FSFI-d	*n*	29	31	15	28	10	24	8	24
Total	Mean (SD)	16.79 (7.38)	17.24 (6.62)	19.12 (7.58)	16.57 (8.56)	16.27 (9.73)	17.86 (9.2)	18.9 (10.67)	14.19 (8.86)
	Md (IQR)	18.2 (10.6–22.3)	18.1 (13.9–22.2)	20.3 (15.25–24.95)	18.65 (10.13–22.1)	18.05 (7.6–24.3)	20.15 (13.13–23.18)	23.1 (15.45–25.4)	15.15 (4.78–19.3)
Desire	Mean (SD)	2.28 (0.95)	2.59 (1.01)	2.8 (0.87)	2.72 (1.15)	2.16 (0.64)	2.78 (1.33)	2.4 (1.06)	2.38 (1.4)
	Md (IQR)	2.4 (1.2–3)	2.4 (2.1–3)	3 (2.4–3.3)	2.4 (1.8–3.6)	2.4 (1.8–2.4)	3 (1.65–3.6)	2.7 (1.2–3.15)	1.8 (1.2–3.6)
Arousal	Mean (SD)	2.76 (1.57)	3.15 (1.39)	3.52 (1.84)	2.83 (1.69)	2.79 (2.18)	3.02 (1.79)	3.75 (2.34)	2.35 (1.69)
	Md (IQR)	3 (1.5–3.9)	3.3 (2.4–3.9)	3.9 (2.55–4.8)	3.3 (1.43–3.9)	3.15 (0.6–4.12)	3.6 (1.95–4.2)	4.8 (3.38–5.18)	2.25 (1.2–3.3)
Lubrication	Mean (SD)	3.68 (1.85)	3.36 (1.63)	3.76 (1.96)	3.16 (1.96)	2.76 (2.59)	3.21 (2.07)	4.05 (2.6)	2.5 (2.23)
	Md (IQR)	3.9 (2.7–5.4)	3.3 (2.1–4.5)	4.2 (2.85–5.25)	3.9 (1.5–4.8)	3.15 (0–5.02)	3.45 (1.88–4.58)	5.25 (2.92–6)	2.55 (0.22–4.05)
Orgasm	Mean (SD)	3.38 (2.11)	3.14 (1.89)	3.65 (2.13)	2.74 (2.23)	3.48 (2.63)	3.05 (2.16)	3.75 (2.42)	3 (2.5)
	Md (IQR)	3.2 (1.6–6)	3.2 (1.4–4.8)	4.4 (2–5.4)	2.6 (0.9–4.9)	4.8 (0.6–5.6)	3.2 (1.5–5.2)	4.8 (2.7–5.3)	3.8 (0–5.3)
Pain	Mean (SD)	1.61 (1.32)	2.21 (1.55)	1.68 (1.85)	2.36 (2.04)	1.68 (2)	2.5 (1.95)	1.25 (2.38)	1.47 (2.19)
	Md (IQR)	1.6 (0–2.4)	2 (1.4–2.8)	1.6 (0–2.8)	2.4 (0–3.7)	1 (0–3)	2.6 (0.6–4)	0 (0–1)	0 (0–1.8)
Satisfaction	Mean (SD)	3.08 (1.32)	2.79 (1.25)	3.71 (1.75)	2.76 (1.54)	3.4 (1.8)	3.3 (1.81)	3.7 (1.93)	2.5 (1.42)
	Md (IQR)	3.2 (1.6–4.4)	2.4 (1.6–4)	4 (2.6–5.2)	2.6 (1.5–3.6)	3.4 (1.9–5)	3.8 (1.5–4.9)	4.6 (2.1–5.2)	2.4 (1.5–3.3)
SSEI-d	*n*	28	31	15	28	10	24	8	24
Total	Mean (SD)	3.98 (0.63)	3.97 (0.72)	4.19 (0.59)	4.09 (0.71)	4.12 (0.82)	4.35 (0.8)	4.25 (0.95)	4.16 (0.7)
	Md (IQR)	3.96 (3.57–4.37)	4.03 (3.39–4.56)	4.31 (3.77–4.51)	4.04 (3.57–4.69)	4.21 (3.84–4.67)	4.49 (3.94–4.89)	4.17 (3.88–5.01)	4.07 (3.54–4.66)
Skill & Experience	Mean (SD)	3.33 (0.82)	3.24 (1.04)	3.5 (0.88)	3.47 (1.08)	3.54 (0.95)	3.8 (1.17)	3.66 (1.02)	3.51 (1.09)
	Md (IQR)	3.43 (2.57–4)	3.29 (2.43–4)	3.86 (2.93–4.07)	3.64 (2.93–4)	3.86 (2.75–4.29)	3.93 (3–4.68)	3.71 (3.39–4.21)	3.57 (2.75–4.18)
Attractiveness	Mean (SD)	4.23 (1.01)	4.26 (0.98)	4.63 (0.86)	4.36 (1.05)	4.47 (1.13)	4.54 (1.14)	4.7 (1.05)	4.48 (0.97)
	Md (IQR)	4.57 (3.68–4.96)	4.29 (3.57–5.14)	4.86 (4.21–5.14)	4.86 (3.5–5.18)	4.71 (3.79–5.43)	4.93 (3.68–5.43)	4.93 (4.21–5.36)	4.93 (3.82–5.29)
Control	Mean (SD)	4.15 (0.9)	4.17 (1.1)	4.17 (1.01)	4.24 (1.03)	4.2 (1.12)	4.4 (1.08)	4.2 (1.16)	4.3 (1.02)
	Md (IQR)	4.29 (3.54–4.75)	4.29 (3.86–4.86)	4.29 (3.57–5)	4.36 (3.82–4.75)	4.5 (3.46–5.07)	4.57 (3.93–5.14)	3.93 (3.29–5.29)	4.43 (3.54–5.07)
Moral Judgement	Mean (SD)	4.62 (0.64)	4.58 (0.8)	4.67 (0.42)	4.67 (0.87)	4.74 (0.73)	4.82 (0.79)	4.91 (0.83)	4.71 (0.9)
	Md (IQR)	4.71 (4.25–5.07)	4.57 (4–5.29)	4.71 (4.43–4.93)	4.64 (4–5.32)	4.86 (4.5–4.96)	4.93 (4.39–5.25)	5.07 (4.79–5.21)	4.57 (3.96–5.46)
Adaptiveness	Mean (SD)	3.55 (1.03)	3.59 (0.85)	3.96 (0.99)	3.71 (0.75)	3.63 (1.07)	4.19 (0.87)	3.79 (1.26)	3.79 (0.87)
	Md (IQR)	3.36 (2.86–4.14)	3.43 (2.86–4.14)	4.14 (3.43–4.64)	3.64 (3.25–4.29)	3.57 (3.04–4.39)	4.21 (3.57–4.64)	3.71 (2.82–4.68)	3.71 (3.39–4.36)
FSQ	*n*	28	30	15	28	10	23	7	24
Fear of Non-Coital Activity	Mean (SD)	12.25 (3.73)	11.17 (4.31)	11.47 (3.11)	11.54 (4.2)	11.6 (4.6)	9.87 (3.53)	11.71 (5.99)	11.62 (5.16)
	Md (IQR)	11.5 (10–15)	11 (8–14)	11 (9.5–13)	12 (8–14.25)	11 (8.25–14)	10 (6.5–12)	11 (7.5–14.5)	11.5 (6.75–15)
Fear of Coitus	Mean (SD)	9.11 (2.85)	8.6 (3.16)	8.47 (2.77)	8.21 (3.37)	8.4 (3.27)	6.96 (3.04)	8.14 (3.58)	7.67 (3.82)
	Md (IQR)	9 (7.75–10.5)	9 (6.25–11)	8 (7–9)	7.5 (5–11.25)	9.5 (6.5–10)	7 (4.5–9)	8 (7–8.5)	7.5 (4–11.25)

VPCQ	*n*	28	30	15	28	10	23	7	24
Control Cognitions	Mean (SD)	4.26 (1.29)	4.32 (1.55)	4.43 (1.17)	4.64 (1.2)	4.7 (1.25)	4.58 (1.21)	4.68 (1.48)	4.53 (1.49)
	Md (IQR)	4.38 (3.19–5.25)	4.62 (3.25–5.75)	4.5 (4–5.12)	4.62 (3.75–5.75)	5.12 (4.56–5.25)	4.75 (3.88–5.5)	5 (4.25–5.75)	5.25 (3.25–6)
Catastrophic and Pain Cognitions	Mean (SD)	3.15 (1.24)	3.23 (1.48)	2.75 (1.54)	2.85 (1.56)	2.72 (1.76)	2.66 (1.53)	2.83 (2.08)	2.94 (2)
	Md (IQR)	3.1 (2.35–3.85)	3.3 (2.4–4.2)	2.6 (1.6–3.5)	2.8 (1.6–4.2)	2.1 (2–3.4)	2.6 (1.8–3.9)	2.6 (1.3–4.7)	2.9 (1.35–4.8)
Self-Image Cognitions	Mean (SD)	2.01 (0.78)	2.09 (1.15)	2.07 (0.86)	1.73 (0.89)	1.68 (0.66)	1.69 (0.78)	1.83 (0.81)	1.72 (1.08)
	Md (IQR)	2 (1.46–2.33)	1.83 (1.17–3.12)	2.17 (1.42–2.5)	1.5 (1.17–2.21)	1.67 (1.08–2.21)	1.5 (1.25–2.08)	1.67 (1.25–2.42)	1.5 (1–2.33)
Positive Cognitions	Mean (SD)	3.03 (1.23)	2.69 (1.22)	3.05 (0.83)	2.54 (0.98)	2.84 (0.7)	2.79 (1.11)	2.66 (0.89)	2.57 (1.16)
	Md (IQR)	3.3 (2.4–3.8)	2.3 (1.8–3.55)	2.8 (2.6–3.5)	2.4 (1.8–3.4)	2.8 (2.6–3.4)	2.8 (2–3.3)	3 (2.2–3.3)	2.2 (1.6–3.65)
Incompatibility	Mean (SD)	1.62 (1.54)	1.68 (1.71)	1.57 (1.71)	1.66 (1.71)	1.35 (2.01)	1.65 (1.45)	1.07 (1.84)	1.42 (1.62)
	Md (IQR)	1.75 (0–2.5)	1.25 (0–2.88)	1 (0–2.5)	1.75 (0–3.12)	0 (0–2.25)	1.5 (0–3)	0 (0–1.25)	1 (0–2.62)
PFB	*n*	26	24	13	22	9	18	7	19
Total	Mean (SD)	71.73 (12.74)	68.29 (15)	71.46 (11)	68.27 (12.4)	71.56 (18.02)	71.06 (13.83)	75 (13.95)	71.32 (12.04)
	Md (IQR)	72 (66–79.75)	72 (60.5–78.25)	74 (68–79)	67 (58.75–79)	79 (60–86)	72.5 (61.25–81)	78 (73.5–83.5)	69 (64–81.5)
Dispute Behavior	Mean (SD)	3.65 (3.91)	5.96 (6.99)	3.31 (3.33)	4.91 (4.71)	3 (3.91)	4.11 (5.26)	3.14 (3.72)	3.74 (4.08)
	Md (IQR)	2 (0.25–5.75)	4 (1.75–7.5)	3 (1–3)	2.5 (2–6)	1 (0–6)	2 (0.25–6.5)	2 (1–3.5)	2 (0.5–6)
Tenderness	Mean (SD)	22.38 (5.94)	21.21 (5.57)	22.08 (5.47)	20.55 (5.03)	21.67 (8.32)	21.67 (6.6)	23.43 (6)	21.74 (5.14)
	Md (IQR)	22.5 (20–27)	21.5 (18.75–25)	23 (22–26)	20 (17.25–25)	25 (16–27)	21 (19–27.75)	23 (22–27.5)	22 (17.5–26)
Communication	Mean (SD)	23 (5.46)	23.04 (4.83)	22.69 (5.14)	22.64 (4.72)	22.89 (6.25)	23.5 (5.07)	24.71 (5.65)	23.32 (4.89)
	Md (IQR)	23.5 (18.75–28)	25 (18.75–26)	22 (19–28)	23.5 (19–27)	23 (20–29)	25.5 (19–27)	26 (21.5–29.5)	22 (19–28)
RSP	*n*	28	30	15	28	10	23	8	24
Total	Mean (SD)	61.36 (16.58)	67.8 (18.91)	59.27 (19.08)	64.54 (17.12)	64.7 (24.06)	63.74 (22.54)	62.38 (27.12)	64.62 (17.74)
	Md (IQR)	59.5 (52.75–73)	71 (52.25–81)	53 (44.5–74)	64.5 (53.75–77.5)	55.5 (46.25–85)	67 (46.5–76.5)	58 (39–79.5)	62.5 (50.75–75.25)
Body Perception	Mean (SD)	13.11 (4.47)	13.27 (4.8)	11.93 (4.42)	12.32 (4.94)	13.8 (5.55)	12.43 (5.89)	11.62 (5.8)	12.29 (4.78)
	Md (IQR)	13 (10–16.5)	13.5 (10–17)	11 (9.5–14.5)	13 (9–15.25)	13 (9.5–16.5)	11 (9–16)	10.5 (7.5–14.5)	10 (9–16.25)
Tenderness	Mean (SD)	9 (3.92)	12.07 (5.7)	9 (4.5)	10.89 (4.52)	10 (6.43)	11.04 (5.61)	9.5 (6.3)	11 (5.6)
	Md (IQR)	8.5 (6–11)	12 (7–15)	7 (7–9.5)	11 (7–13.25)	7 (6–12.5)	10 (6–14)	7 (5–10.75)	10 (6–13.5)
Desire	Mean (SD)	17.86 (5.25)	17.6 (4.1)	15.4 (5.6)	17.64 (4.49)	18.5 (4.77)	17.04 (4.85)	18 (7.07)	18 (4.43)
	Md (IQR)	18 (15.25–21.5)	17 (15–20.75)	15 (11.5–20)	17.5 (14.75–21)	18 (16–23)	15 (14–20.5)	19.5 (10.75–25)	17 (14.75–22)
Love	Mean (SD)	8.79 (2.74)	10.67 (4.4)	8.67 (2.53)	9.93 (3.64)	8.3 (2.87)	9.22 (3.54)	7.75 (2.66)	9.38 (3.7)
	Md (IQR)	9 (6.75–10)	11 (6.25–13.75)	9 (7–10)	10 (6.75–12.25)	7.5 (6–10)	10 (5–11)	7.5 (5.75–9)	9 (6–11.5)
Communication	Mean (SD)	12.61 (4.66)	14.2 (4.78)	14.27 (5.12)	13.75 (4.99)	14.1 (6.76)	14 (6.35)	15.5 (8.21)	13.96 (6.05)
	Md (IQR)	13 (10–15)	15 (10.25–17)	14 (11–16.5)	14 (11.75–16.25)	12.5 (9.25–19.5)	14 (9.5–17.5)	14.5 (8.75–23.5)	13 (9.75–17.5)
CSI-GE	*n*	29	31	15	28	10	24	9	25
	Mean (SD)	43.86 (15.23)	42.81 (16.54)	40.8 (15.01)	43.04 (16)	38.4 (14.21)	36 (15.93)	32.11 (23.56)	43.28 (17.7)
	Md (IQR)	47 (35–53)	44 (33.5–54.5)	41 (29.5–51.5)	45.5 (34–55.25)	36.5 (27.5–49.5)	37 (26.25–50)	32 (12–50)	45 (35–53)
**Overall Health**									
BDI-II	*n*	-	-	-	-	10	24	-	-
	Mean (SD)	-	-	-	-	11.5 (8.82)	9.46 (6.3)	-	-
	Md (IQR)	-	-	-	-	8 (5–18.75)	7 (5–12.75)	-	-
GAD-7	*n*	-	-	-	-	10	24	-	-
	Mean (SD)	-	-	-	-	6 (2.49)	4.75 (4.46)	-	-
	Md (IQR)	-	-	-	-	5.5 (4–7)	3.5 (1–8)	-	-

PPS-10	*n*	28	30	15	28	10	23	7	24
	Mean (SD)	20.21 (4.98)	17.97 (5.41)	18.33 (5.67)	18.46 (4.97)	17.2 (6.3)	15.61 (5.75)	18.29 (8.12)	17.12 (5.86)
	Md (IQR)	19.5 (17–23.5)	18.5 (14–21.75)	18 (13.5–21.5)	19 (15.5–22.25)	16.5 (12.5–20.75)	15 (11–19.5)	17 (14.5–22.5)	16 (14–20)
PROMIS-29	*n*	28	30	15	28	10	23	8	24
Depression	Mean (SD)	61.1 (8.57)	58.27 (9.27)	60.77 (7.66)	56.98 (8.77)	55.57 (10.8)	50.87 (9.49)	59.67 (11.07)	55.79 (9.31)
	Md (IQR)	62.5 (58.35–66.2)	58.9 (53.9–64.4)	61 (56.7–65.3)	56.7 (52.67–62.98)	56.7 (49–60.48)	53.9 (41–56.7)	58.85 (53.9–65.17)	57.8 (52.67–61)
Anxiety	Mean (SD)	58.74 (7.72)	55.1 (7.9)	59.56 (7.08)	55.91 (8.95)	52.35 (7.5)	50.63 (9.26)	57.36 (9.06)	57.27 (7.66)
	Md (IQR)	59.5 (55.27–65.3)	55.8 (51.2–59.5)	59.5 (53.7–64.35)	57.1 (51.2–63.4)	53.7 (49.42–56.25)	51.2 (40.3–55.8)	57.1 (55.62–61.45)	56.45 (51.2–61.5)
Physical Function	Mean (SD)	53.29 (5.35)	53.98 (4.2)	52.61 (6.12)	53.3 (4.48)	53.66 (4.79)	53.72 (4.42)	50.52 (7.58)	52.17 (7.24)
	Md (IQR)	56.9 (51.62–56.9)	56.9 (50.28–56.9)	56.9 (48.45–56.9)	56.9 (49.6–56.9)	56.9 (52.3–56.9)	56.9 (50.95–56.9)	52.1 (46.8–56.9)	56.9 (47.3–56.9)
Pain Interference	Mean (SD)	60.07 (6.97)	57.56 (7)	56.48 (7.59)	54.58 (7.03)	53.38 (11.07)	55.02 (7.5)	50.84 (10.5)	55.57 (7.64)
	Md (IQR)	60.55 (55.6–64.5)	57.1 (55.6–62.17)	55.6 (55.6–61.2)	55.6 (51.4–59.17)	54.55 (41.6–63.88)	55.6 (51.75–59.9)	48.6 (41.6–57.33)	55.6 (53.42–61.2)
Pain Intensity	Mean (SD)	5.07 (1.9)	4.5 (2.08)	4.53 (2.2)	4 (2.02)	3 (2.21)	4.35 (2.08)	2.62 (2.72)	4 (2.54)
	Md (IQR)	5 (4–7)	5 (3–6)	4 (3–6.5)	3.5 (2–5.25)	2.5 (1.25–4.75)	5 (3–5)	2 (0.75–3.5)	5 (1.75–6)
Fatigue	Mean (SD)	64.05 (8.01)	62.84 (10.13)	62.93 (8.43)	59.76 (12.51)	58.13 (9.86)	58.04 (7.79)	65.25 (9.49)	58.98 (9.7)
	Md (IQR)	65.45 (57–71.6)	66.2 (58.7–68.8)	66.2 (56.85–68.8)	64.7 (51.37–66.85)	56.85 (51.8–64.65)	58.7 (51.8–63.2)	67.5 (56.5–72.65)	58.7 (51.8–65.08)
Sleep disturbance	Mean (SD)	56.95 (7.46)	56.01 (8.87)	54.86 (11.55)	56.47 (8.12)	54.97 (8.98)	56.42 (7.35)	54.35 (6.27)	58.3 (7.16)
	Md (IQR)	56.1 (52.3–62.38)	56.1 (50.4–61.9)	54.3 (48.4–63.35)	58.95 (50.4–61.9)	56.1 (48.4–59.48)	54.3 (50.4–61.9)	53.3 (49.9–55.72)	57.9 (51.82–63.5)
Social participation	Mean (SD)	46.02 (6.02)	48.9 (7.83)	47.25 (5.79)	49.71 (7.08)	47.79 (5.27)	49.24 (6.61)	48.9 (5.99)	47.45 (7)
	Md (IQR)	44.9 (42.3–50.6)	46.8 (43.9–48.7)	47.7 (42.3–52.7)	47.7 (45.4–53.45)	47.7 (44.4–52.18)	47.7 (44.9–52.7)	50.7 (46.85–52.7)	46.8 (43.9–48.7)
EUROHIS-QOL	*n*	29	31	15	28	10	24	9	25
	Mean (SD)	25.59 (6.6)	25.16 (6.88)	28.47 (4.53)	27.5 (4.99)	29 (6.27)	25.5 (7.31)	26 (11.2)	26.72 (7.68)
	Md (IQR)	25 (22–30)	25 (22.5–29)	28 (26–31)	27 (23.75–31)	30 (23.75–33)	25 (22–30)	30 (24–31)	27 (25–32)
ISIQ-FLUTS	*n*	28	30	15	28	10	23	7	24
Total	Mean (SD)	8.29 (4.3)	7.4 (5.05)	7 (4.78)	7 (5.67)	6.2 (4.57)	5.35 (4.04)	6 (4.47)	7.75 (5.39)
	Md (IQR)	7.5 (6–11)	7 (4–11)	6 (5–10.5)	7 (3.75–10)	6 (2.25–9.25)	5 (3–6.5)	5 (3–10)	7 (3.75–10)
Filling symptoms	Mean (SD)	4.18 (2.2)	3.87 (2.43)	3.67 (2.61)	3.79 (2.39)	3.2 (2.66)	2.91 (1.56)	3.29 (2.14)	3.75 (2.36)
	Md (IQR)	4 (2–6)	4 (2–5.75)	4 (2–5)	4 (2–5.25)	2.5 (1–5)	3 (2–4)	4 (2–4.5)	3 (2–5.25)
Voiding symptoms	Mean (SD)	2.61 (2.36)	2 (1.95)	2.53 (3)	1.93 (1.94)	2.4 (3.37)	1.48 (1.86)	1.71 (2.06)	2.25 (2.69)
	Md (IQR)	2 (1–4)	2 (0–3)	2 (0.5–3)	1.5 (0–3)	1.5 (0–3)	1 (0–2.5)	1 (0–3)	1 (0–5)
Incontinence symptoms	Mean (SD)	1.5 (2.69)	1.53 (2.49)	0.8 (1.57)	1.29 (2.98)	0.6 (1.26)	0.96 (2.36)	1 (2.24)	1.75 (3.37)
	Md (IQR)	0 (0–1.25)	0 (0–2.75)	0 (0–1)	0 (0–2)	0 (0–0.75)	0 (0–1)	0 (0–0.5)	0 (0–3)

Abbreviation: T0, baseline. T1, after Module 5/5 weeks after baseline. T2, after Module 8/8 weeks after baseline. T3, 6-month follow-up after baseline. IG, intervention group. CG, control group. FSDS-DAO, Female Sexual Distress Scale- Desire/Arousal/Orgasm. FSFI, Female Sexual Function Index. SSEI-d, Sexual Self-Esteem-Inventory (German version). FSQ, Fear of Sexuality Questionnaire. VPCQ, Vaginal Penetration Cognition Questionnaire. PFB, Partnership Questionnaire. RSP, Resources in Sexuality and Partnership. CSI-GE, Central Sensitization Inventory (German version). BDI-II, Beck-Depression-Inventory-II. GAD-7, Generalized Anxiety Disorder Scale-7. PPS-10, Perceived Stress Scale. PROMIS-29, Patient-Reported Outcomes Measurement Information System-29. EUROHIS-QOL, European Health Interview Survey-Quality of Life. ISIQ-FLUTS, International Consultation on Incontinence Questionnaire Female Lower Urinary Tract Symptoms Modules.

Table S12. Baseline-Adjusted Mean Changes and Between-Group Differences (ANCOVA).

		T1-T0				T2-T0				T3-T0			
	**Group**	**n**	**Δ (SD)**	**CI 95%**	***d***	**n**	**Δ (SD)**	**CI 95%**	***d***	**n**	**Δ (SD)**	**CI 95%**	***d***
**Sexual Health Outcomes**													
FSDS-DAO	IG	15	-10.39 (15.24)	[-15.57, -5.22]	-	10	-12.61 (12.76)	[-18.95, -6.27]	-	9	-14.98 (17.89)	[-21.66, -8.29]	-
	CG	28	-3.68 (10.45)	[-7.47, 0.11]	-	24	-14.83 (11.60)	[-18.92, -10.73]	-	25	-6.92 (12.45)	[-11.02, -2.83]	-
	IG vs. CG	-	-6.71 (–)	[-13.13, -0.29]	-0.66	-	2.22 (–)	[-5.33, 9.77]	0.22	-	-8.05 (–)	[-15.89, -0.22]	-0.79
FSFI-d													
Total	IG	15	2.87 (6.97)	[-0.44, 6.19]	-	10	1.29 (9.71)	[-2.78, 5.36]	-	9	3.63 (9.85)	[-0.66, 7.93]	-
	CG	28	-0.45 (7.85)	[-2.87, 1.98]	-	24	0.22 (8.31)	[-2.41, 2.84]	-	25	-2.88 (8.75)	[-5.50, -0.26]	-
	IG vs. CG	-	3.32 (–)	[-0.78, 7.43]	0.51	-	1.07 (–)	[-3.79, 5.92]	0.16	-	6.51 (–)	[1.48, 11.55]	1.00
Desire	IG	15	0.58 (1.05)	[0.16, 1.00]	-	10	0.13 (0.64)	[-0.39, 0.64]	-	9	0.41 (1.14)	[-0.13, 0.95]	-
	CG	28	0.15 (1.14)	[-0.16, 0.45]	-	24	0.13 (1.05)	[-0.20, 0.46]	-	25	-0.15 (1.01)	[-0.48, 0.18]	-
	IG vs. CG	-	0.43 (–)	[-0.08, 0.95]	0.53	-	-0.01 (–)	[-0.62, 0.61]	-0.01	-	0.56 (–)	[-0.08, 1.19]	0.69
Arousal	IG	15	0.64 (2.02)	[-0.12, 1.40]	-	10	0.09 (2.48)	[-0.85, 1.02]	-	9	0.93 (2.16)	[-0.05, 1.92]	-
	CG	28	-0.24 (1.79)	[-0.80, 0.31]	-	24	-0.12 (2.10)	[-0.72, 0.48]	-	25	-0.70 (1.93)	[-1.30, -0.10]	-
	IG vs. CG	-	0.89 (–)	[-0.06, 1.83]	0.59	-	0.21 (–)	[-0.91, 1.32]	0.14	-	1.63 (–)	[0.48, 2.79]	1.09
Lubrication	IG	15	0.09 (1.71)	[-0.66, 0.83]	-	10	-0.75 (2.42)	[-1.66, 0.15]	-	9	0.39 (2.42)	[-0.56, 1.35]	-
	CG	28	-0.08 (1.15)	[-0.62, 0.46]	-	24	-0.10 (1.79)	[-0.68, 0.49]	-	25	-0.69 (2.18)	[-1.28, -0.10]	-
	IG vs. CG	-	0.17 (–)	[-0.75, 1.09]	0.12	-	-0.66 (–)	[-1.74, 0.43]	-0.45	-	1.08 (–)	[-0.04, 2.20]	0.74
Orgasm	IG	15	0.65 (1.55)	[-0.20, 1.50]	-	10	0.84 (2.30)	[-0.20, 1.88]	-	9	0.89 (1.74)	[-0.21, 1.98]	-
	CG	28	-0.39 (2.43)	[-1.01, 0.23]	-	24	-0.26 (1.89)	[-0.93, 0.41]	-	25	-0.26 (2.39)	[-0.93, 0.41]	-
	IG vs. CG	-	1.04 (–)	[-0.01, 2.09]	0.63	-	1.10 (–)	[-0.14, 2.34]	0.66	-	1.15 (–)	[-0.14, 2.43]	0.69
Pain	IG	15	0.17 (1.78)	[-0.59, 0.92]	-	10	0.20 (2.34)	[-0.73, 1.12]	-	9	0.05 (2.65)	[-0.92, 1.03]	-
	CG	28	0.17 (1.68)	[-0.38, 0.72]	-	24	0.18 (1.59)	[-0.42, 0.78]	-	25	-0.75 (1.95)	[-1.35, -0.16]	-
	IG vs. CG	-	-0.00 (–)	[-0.94, 0.94]	-0.00	-	0.02 (–)	[-1.09, 1.13]	0.01	-	0.81 (–)	[-0.34, 1.96]	0.55
Satisfaction	IG	15	0.71 (0.84)	[0.12, 1.29]	-	10	0.75 (1.59)	[0.03, 1.47]	-	9	0.92 (1.39)	[0.16, 1.68]	-
	CG	28	-0.02 (1.60)	[-0.45, 0.41]	-	24	0.41 (1.52)	[-0.05, 0.87]	-	25	-0.30 (1.53)	[-0.76, 0.17]	-
	IG vs. CG	-	0.72 (–)	[-0.00, 1.45]	0.63	-	0.34 (–)	[-0.52, 1.20]	0.29	-	1.22 (–)	[0.33, 2.11]	1.06
SSEI-d													
Total	IG	15	0.13 (0.43)	[-0.04, 0.31]	-	10	0.12 (0.44)	[-0.10, 0.33]	-	9	0.21 (0.66)	[-0.02, 0.43]	-
	CG	28	0.10 (0.31)	[-0.02, 0.23]	-	24	0.32 (0.38)	[0.18, 0.46]	-	25	0.12 (0.49)	[-0.01, 0.26]	-
	IG vs. CG	-	0.03 (–)	[-0.18, 0.25]	0.10	-	-0.20 (–)	[-0.45, 0.05]	-0.59	-	0.08 (–)	[-0.18, 0.35]	0.25
Skill & experience	IG	15	0.21 (0.50)	[-0.04, 0.47]	-	10	0.32 (0.47)	[0.01, 0.64]	-	9	0.39 (0.80)	[0.06, 0.72]	-
	CG	28	0.24 (0.50)	[0.05, 0.43]	-	24	0.50 (0.74)	[0.30, 0.70]	-	25	0.17 (0.70)	[-0.03, 0.37]	-
	IG vs. CG	-	-0.03 (–)	[-0.34, 0.29]	-0.06	-	-0.18 (–)	[-0.55, 0.19]	-0.36	-	0.22 (–)	[-0.17, 0.61]	0.44
Attractiveness	IG	15	0.10 (0.48)	[-0.14, 0.34]	-	10	0.06 (0.39)	[-0.23, 0.36]	-	9	0.14 (0.67)	[-0.17, 0.45]	-
	CG	28	0.03 (0.59)	[-0.15, 0.20]	-	24	0.19 (0.65)	[-0.00, 0.37]	-	25	0.14 (0.63)	[-0.05, 0.33]	-
	IG vs. CG	-	0.07 (–)	[-0.22, 0.37]	0.15	-	-0.12 (–)	[-0.47, 0.23]	-0.26	-	-0.01 (–)	[-0.37, 0.36]	-0.01
Moral judgement	IG	15	-0.03 (0.63)	[-0.26, 0.19]	-	10	-0.01 (0.52)	[-0.29, 0.27]	-	9	0.17 (0.44)	[-0.12, 0.47]	-
	CG	28	0.10 (0.54)	[-0.07, 0.27]	-	24	0.17 (0.57)	[-0.01, 0.35]	-	25	0.05 (0.66)	[-0.13, 0.23]	-
	IG vs. CG	-	-0.13 (–)	[-0.42, 0.15]	-0.30	-	-0.17 (–)	[-0.51, 0.16]	-0.39	-	0.12 (–)	[-0.23, 0.47]	0.27
Control	IG	15	0.01 (0.86)	[-0.31, 0.33]	-	10	0.03 (0.96)	[-0.36, 0.42]	-	9	0.07 (1.13)	[-0.34, 0.48]	-

	CG	28	0.07 (0.68)	[-0.16, 0.31]	-	24	0.26 (0.67)	[0.01, 0.51]	-	25	0.14 (0.86)	[-0.11, 0.39]	-
	IG vs. CG	-	-0.06 (–)	[-0.46, 0.33]	-0.10	-	-0.23 (–)	[-0.69, 0.23]	-0.37	-	-0.07 (–)	[-0.55, 0.41]	-0.11
Adaptiveness	IG	15	0.38 (0.83)	[0.09, 0.67]	-	10	0.16 (0.87)	[-0.20, 0.52]	-	9	0.25 (1.11)	[-0.13, 0.63]	-
	CG	28	0.07 (0.56)	[-0.14, 0.28]	-	24	0.49 (0.69)	[0.26, 0.72]	-	25	0.12 (0.77)	[-0.11, 0.35]	-
	IG vs. CG	-	0.31 (–)	[-0.05, 0.67]	0.55	-	-0.33 (–)	[-0.75, 0.10]	-0.58	-	0.13 (–)	[-0.31, 0.57]	0.23
FSQ													
Fear of Coitus	IG	15	-0.60 (1.72)	[-1.59, 0.39]	-	10	-1.13 (2.91)	[-2.34, 0.09]	-	9	-1.11 (2.49)	[-2.47, 0.24]	-
	CG	28	-0.18 (2.22)	[-0.91, 0.54]	-	24	-1.46 (2.62)	[-2.26, -0.66]	-	25	-0.84 (2.90)	[-1.62, -0.06]	-
	IG vs. CG	-	-0.41 (–)	[-1.64, 0.81]	-0.21	-	0.33 (–)	[-1.12, 1.79]	0.17	-	-0.27 (–)	[-1.84, 1.29]	-0.14
Fear of Non-Coital Activity	IG	15	-0.81 (3.83)	[-2.31, 0.69]	-	10	-1.26 (4.19)	[-3.11, 0.59]	-	9	-1.70 (4.40)	[-3.77, 0.37]	-
	CG	28	0.66 (3.57)	[-0.44, 1.76]	-	24	-0.85 (3.63)	[-2.07, 0.37]	-	25	0.76 (4.04)	[-0.43, 1.95]	-
	IG vs. CG	-	-1.47 (–)	[-3.34, 0.40]	-0.50	-	-0.41 (–)	[-2.63, 1.82]	-0.14	-	-2.46 (–)	[-4.86, -0.06]	-0.84
VPCQ													
Control Cognitions	IG	15	0.04 (1.38)	[-0.42, 0.50]	-	10	0.39 (0.96)	[-0.17, 0.95]	-	9	0.62 (0.97)	[-0.01, 1.25]	-
	CG	28	0.25 (1.30)	[-0.09, 0.58]	-	24	0.24 (1.20)	[-0.13, 0.61]	-	25	0.19 (1.39)	[-0.17, 0.55]	-
	IG vs. CG	-	-0.20 (–)	[-0.77, 0.37]	-0.22	-	0.15 (–)	[-0.53, 0.82]	0.17	-	0.43 (–)	[-0.30, 1.16]	0.48
Catastrophic and Pain Cognitions	IG	15	-0.50 (1.40)	[-1.02, 0.01]	-	10	-0.55 (1.02)	[-1.19, 0.08]	-	9	-0.53 (1.40)	[-1.24, 0.18]	-
	CG	28	-0.31 (1.02)	[-0.69, 0.07]	-	24	-0.57 (1.36)	[-0.98, -0.15]	-	25	-0.36 (1.43)	[-0.77, 0.05]	-
	IG vs. CG	-	-0.19 (–)	[-0.84, 0.45]	-0.19	-	0.01 (–)	[-0.75, 0.77]	0.01	-	-0.17 (–)	[-0.99, 0.65]	-0.17
Self-Image Cognitions	IG	15	0.14 (0.71)	[-0.13, 0.42]	-	10	-0.18 (0.56)	[-0.52, 0.15]	-	9	0.05 (0.39)	[-0.33, 0.43]	-
	CG	28	-0.26 (0.72)	[-0.46, -0.06]	-	24	-0.21 (0.87)	[-0.43, 0.01]	-	25	-0.24 (0.74)	[-0.45, -0.02]	-
	IG vs. CG	-	0.40 (–)	[0.06, 0.74]	0.75	-	0.03 (–)	[-0.37, 0.43]	0.05	-	0.28 (–)	[-0.15, 0.72]	0.53
Incompatibility	IG	15	-0.27 (0.88)	[-0.68, 0.13]	-	10	-0.52 (0.86)	[-1.02, -0.02]	-	9	-0.85 (0.99)	[-1.41, -0.29]	-
	CG	28	0.00 (0.98)	[-0.30, 0.30]	-	24	0.15 (1.04)	[-0.18, 0.48]	-	25	-0.14 (1.20)	[-0.46, 0.18]	-
	IG vs. CG	-	-0.27 (–)	[-0.78, 0.23]	-0.34	-	-0.67 (–)	[-1.27, -0.07]	-0.84	-	-0.71 (–)	[-1.35, -0.06]	-0.89
Positive Cognitions	IG	15	0.03 (0.90)	[-0.35, 0.42]	-	10	-0.03 (0.72)	[-0.50, 0.44]	-	9	-0.08 (0.59)	[-0.60, 0.44]	-
	CG	28	-0.22 (1.26)	[-0.50, 0.06]	-	24	0.02 (0.91)	[-0.29, 0.33]	-	25	-0.16 (1.20)	[-0.46, 0.14]	-
	IG vs. CG	-	0.25 (–)	[-0.23, 0.73]	0.33	-	-0.05 (–)	[-0.61, 0.51]	-0.07	-	0.08 (–)	[-0.52, 0.68]	0.11
PFB													
Total	IG	14	-0.33 (7.39)	[-4.54, 3.89]	-	9	-0.23 (12.05)	[-5.30, 4.83]	-	8	-0.34 (14.37)	[-5.73, 5.05]	-
	CG	23	-1.91 (8.46)	[-5.14, 1.33]	-	19	0.18 (8.23)	[-3.40, 3.76]	-	21	1.00 (11.71)	[-2.48, 4.49]	-
	IG vs. CG	-	1.58 (–)	[-3.74, 6.89]	0.21	-	-0.42 (–)	[-6.62, 5.79]	-0.05	-	-1.34 (–)	[-7.77, 5.08]	-0.18
Disruptive behavior	IG	14	0.28 (3.06)	[-1.47, 2.03]	-	9	0.04 (3.47)	[-2.07, 2.14]	-	8	0.34 (4.26)	[-1.90, 2.57]	-
	CG	23	0.28 (4.67)	[-1.07, 1.62]	-	19	-0.14 (4.77)	[-1.62, 1.34]	-	21	-0.76 (4.99)	[-2.21, 0.68]	-
	IG vs. CG	-	0.00 (–)	[-2.21, 2.21]	0.00	-	0.18 (–)	[-2.40, 2.75]	0.06	-	1.10 (–)	[-1.56, 3.76]	0.35
Tenderness	IG	14	0.25 (3.59)	[-1.76, 2.25]	-	9	0.06 (6.90)	[-2.35, 2.48]	-	8	-0.24 (7.87)	[-2.80, 2.33]	-
	CG	23	-1.02 (3.46)	[-2.56, 0.53]	-	19	0.02 (3.62)	[-1.69, 1.73]	-	21	0.19 (5.43)	[-1.47, 1.86]	-
	IG vs. CG	-	1.26 (–)	[-1.27, 3.80]	0.35	-	0.04 (–)	[-2.91, 3.00]	0.01	-	-0.43 (–)	[-3.49, 2.63]	-0.12
Communication	IG	14	-0.06 (3.76)	[-1.42, 1.31]	-	9	-0.04 (3.24)	[-1.67, 1.60]	-	8	0.47 (4.31)	[-1.26, 2.21]	-
	CG	23	-0.71 (2.85)	[-1.76, 0.33]	-	19	-0.02 (2.29)	[-1.18, 1.14]	-	21	-0.04 (3.29)	[-1.16, 1.09]	-
	IG vs. CG	-	0.66 (–)	[-1.06, 2.37]	0.26	-	-0.02 (–)	[-2.02, 1.99]	-0.01	-	0.51 (–)	[-1.56, 2.58]	0.21
RSP													
Total	IG	15	-3.88 (12.93)	[-9.38, 1.61]	-	10	-1.84 (15.02)	[-8.57, 4.88]	-	9	-1.12 (17.18)	[-8.21, 5.97]	-
	CG	28	-1.99 (11.05)	[-6.01, 2.03]	-	24	-2.80 (10.38)	[-7.23, 1.64]	-	25	-2.31 (17.58)	[-6.66, 2.03]	-

	IG vs. CG	-	-1.90 (–)	[-8.71, 4.92]	-0.18	-	0.96 (–)	[-7.10, 9.01]	0.09	-	1.19 (–)	[-7.12, 9.51]	0.11
Body perception	IG	15	-1.13 (3.98)	[-2.52, 0.25]	-	10	0.20 (4.09)	[-1.51, 1.90]	-	9	-0.92 (4.14)	[-2.71, 0.88]	-
	CG	28	-0.72 (2.95)	[-1.74, 0.30]	-	24	-0.50 (3.03)	[-1.62, 0.62]	-	25	-0.54 (3.68)	[-1.64, 0.56]	-
	IG vs. CG	-	-0.41 (–)	[-2.13, 1.31]	-0.15	-	0.70 (–)	[-1.34, 2.73]	0.26	-	-0.37 (–)	[-2.48, 1.73]	-0.14
Desire	IG	15	-2.76 (5.79)	[-4.66, -0.85]	-	10	-0.64 (4.61)	[-2.98, 1.71]	-	9	-0.79 (7.20)	[-3.26, 1.69]	-
	CG	28	-0.02 (4.25)	[-1.41, 1.38]	-	24	-0.49 (4.56)	[-2.03, 1.05]	-	25	0.25 (4.74)	[-1.25, 1.76]	-
	IG vs. CG	-	-2.74 (–)	[-5.10, -0.38]	-0.73	-	-0.15 (–)	[-2.96, 2.66]	-0.04	-	-1.04 (–)	[-3.94, 1.86]	-0.28
Tenderness	IG	15	-0.37 (2.04)	[-1.75, 1.00]	-	10	-0.10 (5.15)	[-1.78, 1.58]	-	9	-0.26 (3.57)	[-2.03, 1.50]	-
	CG	28	-0.73 (2.81)	[-1.73, 0.27]	-	24	-0.68 (2.44)	[-1.79, 0.43]	-	25	-0.80 (4.98)	[-1.88, 0.29]	-
	IG vs. CG	-	0.36 (–)	[-1.35, 2.07]	0.13	-	0.58 (–)	[-1.43, 2.60]	0.22	-	0.53 (–)	[-1.55, 2.61]	0.20
Love	IG	15	-0.46 (2.62)	[-1.55, 0.63]	-	10	-0.78 (1.35)	[-2.11, 0.55]	-	9	-0.52 (2.26)	[-1.92, 0.88]	-
	CG	28	-0.27 (3.16)	[-1.07, 0.52]	-	24	-0.92 (2.74)	[-1.79, -0.04]	-	25	-0.84 (3.66)	[-1.70, 0.02]	-
	IG vs. CG	-	-0.19 (–)	[-1.53, 1.16]	-0.09	-	0.14 (–)	[-1.46, 1.73]	0.06	-	0.32 (–)	[-1.33, 1.97]	0.15
Communication	IG	15	0.69 (2.66)	[-1.13, 2.51]	-	10	-0.41 (5.56)	[-2.64, 1.82]	-	9	1.46 (5.50)	[-0.89, 3.81]	-
	CG	28	-0.20 (3.38)	[-1.53, 1.13]	-	24	-0.21 (3.71)	[-1.68, 1.26]	-	25	-0.32 (6.04)	[-1.76, 1.12]	-
	IG vs. CG	-	0.89 (–)	[-1.36, 3.15]	0.25	-	-0.20 (–)	[-2.86, 2.47]	-0.05	-	1.78 (–)	[-0.98, 4.53]	0.50
CSI-GE	IG	15	-2.43 (9.26)	[-8.27, 3.41]	-	10	-3.45 (7.97)	[-10.61, 3.71]	-	9	-4.75 (13.83)	[-12.30, 2.79]	-
	CG	28	-0.67 (13.37)	[-4.95, 3.60]	-	24	-7.11 (12.90)	[-11.73, -2.49]	-	25	-1.30 (21.70)	[-5.83, 3.23]	-
	IG vs. CG	-	-1.76 (–)	[-9.00, 5.48]	-0.15	-	3.66 (–)	[-4.86, 12.18]	0.32	-	-3.45 (–)	[-12.26, 5.36]	-0.30
**Overall Health Outcomes**													
PSS-10	IG	15	-0.46 (3.95)	[-2.65, 1.73]	-	10	-1.41 (3.84)	[-4.08, 1.27]	-	9	0.72 (6.12)	[-2.28, 3.71]	-
	CG	28	0.75 (5.26)	[-0.85, 2.35]	-	24	-1.55 (5.91)	[-3.32, 0.23]	-	25	-0.15 (6.97)	[-1.89, 1.58]	-
	IG vs. CG	-	-1.21 (–)	[-3.92, 1.50]	-0.28	-	0.14 (–)	[-3.08, 3.35]	0.03	-	0.87 (–)	[-2.59, 4.33]	0.20
PROMIS-29													
Anxiety	IG	15	2.89 (6.91)	[-0.22, 6.01]	-	10	-3.90 (10.28)	[-7.71, -0.09]	-	9	1.52 (8.26)	[-2.50, 5.54]	-
	CG	28	0.91 (7.04)	[-1.37, 3.20]	-	24	-4.53 (7.82)	[-7.05, -2.02]	-	25	2.21 (9.74)	[-0.25, 4.68]	-
	IG vs. CG	-	1.98 (–)	[-1.89, 5.85]	0.32	-	0.63 (–)	[-3.94, 5.20]	0.10	-	-0.69 (–)	[-5.41, 4.02]	-0.11
Depression	IG	15	1.52 (8.37)	[-1.88, 4.91]	-	10	-2.21 (8.04)	[-6.37, 1.95]	-	9	3.83 (8.33)	[-0.57, 8.22]	-
	CG	28	-0.86 (7.83)	[-3.34, 1.63]	-	24	-7.07 (9.30)	[-9.81, -4.32]	-	25	-1.89 (10.51)	[-4.57, 0.80]	-
	IG vs. CG	-	2.37 (–)	[-1.84, 6.59]	0.36	-	4.85 (–)	[-0.13, 9.84]	0.73	-	5.71 (–)	[0.57, 10.86]	0.86
Fatigue	IG	15	0.69 (8.98)	[-3.11, 4.49]	-	10	-2.89 (10.54)	[-7.55, 1.78]	-	9	4.79 (9.19)	[-0.13, 9.71]	-
	CG	28	-2.97 (11.30)	[-5.75, -0.19]	-	24	-5.19 (7.73)	[-8.26, -2.12]	-	25	-4.07 (10.57)	[-7.07, -1.06]	-
	IG vs. CG	-	3.67 (–)	[-1.04, 8.37]	0.49	-	2.30 (–)	[-3.28, 7.89]	0.31	-	8.86 (–)	[3.09, 14.63]	1.19
Physical function	IG	15	-1.60 (3.92)	[-3.21, 0.01]	-	10	0.29 (1.77)	[-1.68, 2.26]	-	9	-3.05 (4.63)	[-5.13, -0.97]	-
	CG	28	-0.46 (3.41)	[-1.64, 0.72]	-	24	0.31 (2.96)	[-0.99, 1.61]	-	25	-1.38 (5.35)	[-2.65, -0.11]	-
	IG vs. CG	-	-1.14 (–)	[-3.13, 0.86]	-0.36	-	-0.02 (–)	[-2.38, 2.34]	-0.01	-	-1.67 (–)	[-4.11, 0.76]	-0.53
Sleep disturbance	IG	15	-0.36 (9.14)	[-3.61, 2.89]	-	10	0.60 (6.47)	[-3.38, 4.59]	-	9	2.68 (8.14)	[-1.53, 6.88]	-
	CG	28	0.62 (7.30)	[-1.75, 3.00]	-	24	0.64 (8.31)	[-1.98, 3.26]	-	25	2.47 (11.13)	[-0.09, 5.04]	-
	IG vs. CG	-	-0.98 (–)	[-5.01, 3.04]	-0.15	-	-0.04 (–)	[-4.80, 4.73]	-0.01	-	0.21 (–)	[-4.72, 5.13]	0.03
Social participation	IG	15	0.60 (4.96)	[-2.04, 3.24]	-	10	1.00 (5.59)	[-2.23, 4.22]	-	9	1.82 (6.58)	[-1.59, 5.22]	-
	CG	28	1.10 (8.24)	[-0.83, 3.02]	-	24	0.52 (5.36)	[-1.61, 2.64]	-	25	-1.18 (9.38)	[-3.27, 0.90]	-
	IG vs. CG	-	-0.50 (–)	[-3.77, 2.78]	-0.10	-	0.48 (–)	[-3.39, 4.35]	0.09	-	3.00 (–)	[-1.00, 7.00]	0.58

Pain interference	IG	15	-2.22 (5.88)	[-5.18, 0.74]	-	10	-5.60 (11.02)	[-9.23, -1.97]	-	9	-6.87 (7.95)	[-10.70, -3.05]	-
	CG	28	-2.99 (5.59)	[-5.17, -0.82]	-	24	-3.28 (7.44)	[-5.67, -0.89]	-	25	-2.54 (8.45)	[-4.88, -0.20]	-
	IG vs. CG	-	0.77 (–)	[-2.90, 4.45]	0.13	-	-2.32 (–)	[-6.66, 2.03]	-0.40	-	-4.34 (–)	[-8.82, 0.15]	-0.75
Pain intensity	IG	15	-0.21 (1.58)	[-0.99, 0.57]	-	10	-1.36 (2.36)	[-2.32, -0.41]	-	9	-1.41 (2.29)	[-2.42, -0.41]	-
	CG	28	-0.41 (1.91)	[-0.98, 0.17]	-	24	-0.15 (1.58)	[-0.78, 0.48]	-	25	-0.48 (2.34)	[-1.10, 0.13]	-
	IG vs. CG	-	0.20 (–)	[-0.77, 1.16]	0.13	-	-1.21 (–)	[-2.36, -0.07]	-0.79	-	-0.93 (–)	[-2.11, 0.25]	-0.61
EUROHIS-QOL	IG	15	0.71 (2.85)	[-1.39, 2.82]	-	10	0.48 (3.74)	[-2.10, 3.06]	-	9	-0.45 (4.66)	[-3.18, 2.27]	-
	CG	28	1.00 (3.21)	[-0.54, 2.53]	-	24	-1.31 (7.33)	[-2.97, 0.35]	-	25	0.27 (5.95)	[-1.35, 1.90]	-
	IG vs. CG	-	-0.29 (–)	[-2.89, 2.32]	-0.07	-	1.79 (–)	[-1.28, 4.86]	0.43	-	-0.73 (–)	[-3.90, 2.45]	-0.18
ISIQ-FLUTS													
Total	IG	15	0.49 (2.85)	[-0.89, 1.87]	-	10	-0.40 (2.79)	[-2.09, 1.29]	-	9	-0.50 (3.38)	[-2.39, 1.38]	-
	CG	28	-0.27 (3.89)	[-1.27, 0.74]	-	24	-2.00 (3.13)	[-3.12, -0.89]	-	25	0.26 (3.60)	[-0.82, 1.35]	-
	IG vs. CG	-	0.75 (–)	[-0.95, 2.46]	0.28	-	1.60 (–)	[-0.42, 3.62]	0.59	-	-0.76 (–)	[-2.94, 1.41]	-0.28
Filling symptoms	IG	15	0.44 (1.60)	[-0.21, 1.10]	-	10	-0.16 (1.97)	[-0.96, 0.63]	-	9	-0.52 (2.07)	[-1.41, 0.37]	-
	CG	28	0.07 (1.59)	[-0.41, 0.54]	-	24	-0.75 (1.42)	[-1.28, -0.23]	-	25	0.00 (1.62)	[-0.51, 0.52]	-
	IG vs. CG	-	0.38 (–)	[-0.43, 1.18]	0.29	-	0.59 (–)	[-0.37, 1.54]	0.46	-	-0.52 (–)	[-1.55, 0.51]	-0.41
Voiding symptoms	IG	15	0.00 (1.44)	[-0.79, 0.79]	-	10	0.04 (1.41)	[-0.93, 1.00]	-	9	0.22 (2.10)	[-0.86, 1.29]	-
	CG	28	-0.07 (2.03)	[-0.65, 0.51]	-	24	-0.40 (1.85)	[-1.04, 0.23]	-	25	0.26 (2.37)	[-0.37, 0.88]	-
	IG vs. CG	-	0.07 (–)	[-0.91, 1.05]	0.05	-	0.44 (–)	[-0.71, 1.59]	0.29	-	-0.04 (–)	[-1.29, 1.21]	-0.03
Incontinence symptoms	IG	15	0.11 (0.64)	[-0.45, 0.66]	-	10	-0.22 (0.42)	[-0.90, 0.46]	-	9	-0.14 (0.64)	[-0.90, 0.62]	-
	CG	28	-0.28 (1.61)	[-0.69, 0.12]	-	24	-0.90 (1.44)	[-1.35, -0.45]	-	25	-0.03 (1.60)	[-0.47, 0.40]	-
	IG vs. CG	-	0.39 (–)	[-0.29, 1.08]	0.36	-	0.68 (–)	[-0.13, 1.49]	0.63	-	-0.11 (–)	[-0.98, 0.77]	-0.10

Abbreviation. T0, baseline. T1, after Module 5/5 weeks after baseline. T2, after Module 8/8 weeks after baseline. T3, 6-month follow-up after baseline. IG, intervention group. CG, control group. Δ, Mean group differences. SD, Standard Deviation. *d*, Cohen's *d*. FSDS-DAO, Female Sexual Distress Scale- Desire/Arousal/Orgasm. FSFI, Female Sexual Function Index. SSEI-d, Sexual Self-Esteem-Inventory (German version). FSQ, Fear of Sexuality Questionnaire. VPCQ, Vaginal Penetration Cognition Questionnaire. PFB, Partnership Questionnaire. RSP, Resources in Sexuality and Partnership. CSI-GE, Central Sensitization Inventory (German version). BDI-II, Beck-Depression-Inventory-II. GAD-7, Generalized Anxiety Disorder Scale-7. PPS-10, Perceived Stress Scale. PROMIS-29, Patient-Reported Outcomes Measurement Information System-29. EUROHIS-QOL, European Health Interview Survey-Quality of Life. ISIQ-FLUTS, International Consultation on Incontinence Questionnaire Female Lower Urinary Tract Symptoms Modules.
