# Supplementary material for: Adherence, Acceptability, and Sexual Health Outcomes of the Odeya App–Based Intervention for Sexual Distress in Women With Endometriosis: Randomized Controlled Mixed Methods Trial
Source: J Med Internet Res. 2026 Feb 19;28:e86042. doi: 10.2196/86042 (PMC12963981; doi:10.2196/86042)
Supplement: Multimedia Appendix 3 [file jmir_v28i1e86042_app3.pdf]

Multimedia Appendix 3

Corresponding Author:
Dr. med. Laura Hatzler
Institute of Sexology and Sexual Medicine
Department of Urology
Charité-Universitätsmedizin Berlin
Charité Platz 1, 10117 Berlin, Germany
Phone: +49 30 450 617 139
Fax: +49 30 529 992
E-Mail: laura.hatzler@charite.de

Protocol 1 for Interview with Completers.

Protocol 2 for Interviews with Control Group.

Protocol 3 for Interviews with Dropouts.

Protocol 1 for Interview with Completers

1. **User Experience with the Digital Intervention**

- How did you experience using the app overall?
- Which aspects of using the app did you find particularly positive, challenging, or frustrating?

2. **Perceived Changes from the Intervention**

- What changes have you experienced since you started using the app?
- What changes have you noticed in terms of your sexuality and, if applicable, your relationship since starting to use the app?
- Which changes do you associate with using the app?
    - From when did you notice a noticeable change?

3. **Perceived Effectiveness and Satisfaction**

- How satisfied are you overall with the app (e.g., regarding content, exercises, multimedia elements)?
- Which features, content, or exercises did you find particularly helpful?
- Were there any content, exercises, or features that you felt were missing? If so, which ones?

4. **Open Questions**

- Did you feel that additional support offerings were missing?
    - This could be an extension within the app or a service outside the app.
- Were there any topics you would have liked to discuss here that have not yet been addressed?

Additional Questions:

- You have completed all modules. Others might find it more difficult. What factors could motivate you or other users to use the app long-term?
- What expectations did you have of the app?

<u>Protocol 2 for Interviews with Control Group</u>

1. **Expectations and Benefits of the Intervention**

- How did you experience participating in this study overall?
- What expectations did you have of the app?

2. **Experience with Study Participation / Perception of Access**

- What did it specifically mean for you not to receive access to the intervention?
    - How did it feel (emotions and thoughts) to be excluded from participating in the digital intervention?
- How did you understand access to the intervention during the waiting period? What did you think would happen after the waiting period?

3. **Changes**

- What changes have you experienced since the start of the study in terms of your sexuality or overall well-being?
- Have you developed strategies to cope with sexual challenges during the waiting period?

4. **Alternative Support and Services**

- Have you sought additional support or information on sexual health outside of the study?

5. **Need for and Interest in Future Offerings**

- Is there still a need for this intervention?

Protocol 3 for Interviews with Dropouts

[Instruction:] We will ask you 3 questions. Please name the most relevant aspects for each question in order of importance and then briefly describe what you mean (1–2 sentences). It is important for us to identify as many aspects as possible, up to a maximum of 10, but fewer are also acceptable. If you feel you have mentioned all relevant aspects, we will move on to the next question.

1) What circumstances or aspects led you to stop using the app?

ASPECTS	DESCRIPTION
**1**	
**2**	
**3**	
**4**	
**5**	
**6**	
**7**	
**8**	
**9**	
**10**	

2) In your opinion, what could be done to prevent users from stopping?

ASPECTS	DESCRIPTION
**1**	
**2**	
**3**	
**4**	
**5**	
**6**	
**7**	
**8**	
**9**	
**10**	

3) What other aspects might have motivated you or helped you to continue using the digital intervention?

ASPECTS	DESCRIPTION
1	
2	
3	
4	
5	
6	
7	
8	
9	
10	
